# The Evolutionary History of Protein Domains Viewed by Species Phylogeny

**DOI:** 10.1371/journal.pone.0008378

**Published:** 2009-12-21

**Authors:** Song Yang, Philip E. Bourne

**Affiliations:** Skaggs School of Pharmacy and Pharmaceutical Sciences, University of California San Diego, San Diego, California, United States of America; University of Oxford, United Kingdom

## Abstract

**Background:**

Protein structural domains are evolutionary units whose relationships can be detected over long evolutionary distances. The evolutionary history of protein domains, including the origin of protein domains, the identification of domain loss, transfer, duplication and combination with other domains to form new proteins, and the formation of the entire protein domain repertoire, are of great interest.

**Methodology/Principal Findings:**

A methodology is presented for providing a parsimonious domain history based on gain, loss, vertical and horizontal transfer derived from the complete genomic domain assignments of 1015 organisms across the tree of life. When mapped to species trees the evolutionary history of domains and domain combinations is revealed, and the general evolutionary trend of domain and combination is analyzed.

**Conclusions/Significance:**

We show that this approach provides a powerful tool to study how new proteins and functions emerged and to study such processes as horizontal gene transfer among more distant species.

## Introduction

Originally discovered as spatially distinct regions of proteins, protein domains are now considered discrete evolutionary units. One basic physical property–the ability to fold independently–defines the features of protein domains including their evolutionary significance. As stable 3D structures, each covering significant sequence space, with relationships between those sequences perhaps undetectable by sequence methods, domains have much to offer in the study of evolution. Add to that features of domain arrangements [1] and a limited number relative to the immense possibilities of sequence space [2] and we have the makings of a powerful method of analysis.

Given these properties, protein domains have been used recently in the study of evolution on genome-wide and species-wide scales [3]–[5]. For example, protein domain content (PDC), the mere presence or absence of domains in completely sequenced genomes, has been shown to define the major evolutionary changes that lead to the genome content of contemporary organisms. Stated another way, the phylogenetic tree of life reconstructed based on PDC is comparable to standard phylogenetic methods based on molecular markers (such as rRNA) and other phylogenomic approaches such as gene content and gene order [5]. This ability verifies the evolutionary importance of protein domains.

Since protein domains are major evolutionary units, their evolutionary histories are of great interest [6]. Questions relating to the origin of domains, the identification of domain loss, transfer, duplication and combination with other domains to form new proteins, and the formation of the entire protein domain repertoire [2] remain challenging topics in evolutionary biology. Beyond evolutionary biology, understanding of domain evolution has a role in assigning function to a rapidly increasing body of data associated with proteomics.

Protein domain evolution is already a well studied area. Having started with identifying the distribution of single-domain and multi-domain proteins in the three superkingdoms [7]–[9], the focus shifted to domain duplication [10], the convergence and divergence of protein domains [11], [12], and especially the formation of multi-domain proteins through domain combination [13]–[16]. Three recent studies considered the evolution of multi-domain proteins using phylogenetic information. Fong et al. viewed the domain architecture in multi-domain proteins as the rearrangement of existing architectures and acquisition of new domains, and proposed a parsimony model to represent these evolutionary pathways [17]. Guided by the evolutionary information in phylogenetic trees, Ekman et al. studied the rate of domain architecture formation and found that there are elevated rates of domain rearrangement in Metazoa [18]. Similarly, Itoh et al. observed many group-specific domain combinations in animals and investigated the difference in domain combinations among different phylogenetic groups [19]. These previous studies each focus on specific aspects of protein domain evolution; in this study, we take a more global view, setting the stage for an investigation of the entire evolutionary history of protein domains throughout the tree of life. This implies a consideration of the origin of domains, domain loss, transfer and combination, mapped to the evolutionary history of organisms, specifically the species phylogeny.

We consider the evolution of protein domains as two distinct but related events: changes to the characteristics of a protein domain and changes to the occurrence of the protein domain in the genomes of different organisms. The former includes the innovation of new domains, the gradual change in domain sequence and structure, and the formation of new domain combinations. Although protein domains have stable 3D structures and are more conserved than sequences, progressive fold changes do occur during evolution, resulting in variations in sequence and structure within a superfamily [20], or even the genesis of a new fold [21]. Domain combination and recombination is a major way of creating new proteins and new functions. Although being involved in a new combination will not change a domain immediately, the structural environment and the evolutionary constraints on the domain have changed and this will eventually affect its sequence, structure and function. (Domain pairs in combination can be considered as new structural, functional and evolutionary units at a higher level.) Thus domain combinations also imply changes in the characteristics of the individual domains. The methodology described subsequently implies the detection of the identity of these domains, before and after such changes.

Given the ability to detect these domains, evolutionary domain events, such as duplication, combination, loss and transfer of a domain between species, change the genomic content of domains or domain combinations, but not their identities. The emergence of a new domain in a species depicts the origin of the domain, unless there is evidence of horizontal transfer from a species believed to have evolved earlier. The duplication of a domain induces divergence of the duplicate domain through mutations, insertions or deletions, producing modified structures and functions that distinguish it from its ancestor, but in our methodology it is only identified if it retains detectable structural similarity.

Here we propose an approach that takes full advantage of existing phylogenetic information to derive the entire evolutionary history of each domain throughout the tree of life. First, the evolutionary processes that change the existence of protein domains and domain combinations in each species, such as loss and transfer, are directly obtained from domain trees or combination trees. Then, the changes to domain identity, such as the divergence of a domain superfamily into different families and formation of new combinations of domains, can be inferred.

## Results

### Phylogenetic Tree of Protein Domains and Combinations

In previous studies, a Venn diagram analysis has often been used to show the distribution of protein domains in the three superkingdoms, archaea, bacteria and eukaryotes, thus depicting the number and types of protein domains in the last universal common ancestor of life (LUCA) and their early evolution [4], [5], [22]. The Venn diagram reflects the evolution of protein domains at the root of the tree, where each superkingdom is considered as one single clade. Based on the same idea, a domain tree is the distribution of protein domains (or their combination) in every taxon across the whole tree of life, and from the perspective of protein domains, reflects the entire evolutionary process from LUCA to organisms existing today.

A domain tree is simply constructed by labeling and characterizing each leaf organism of the phylogenetic tree by the type and numbers of protein domains in its genome. Even though no general agreement has been reached about the universal tree of life, the NCBI taxonomy, which is based on extensive genetic and morphological evidence and built by standard molecular phylogenetic methods, is used as the standard species phylogeny in this study. The hierarchical structure of the NCBI-derived phylogenetic tree is identical for every domain; each domain, however, has its own corresponding domain tree, depicting its unique distribution on the species tree and its distinct evolutionary history.

**Figure 1:**
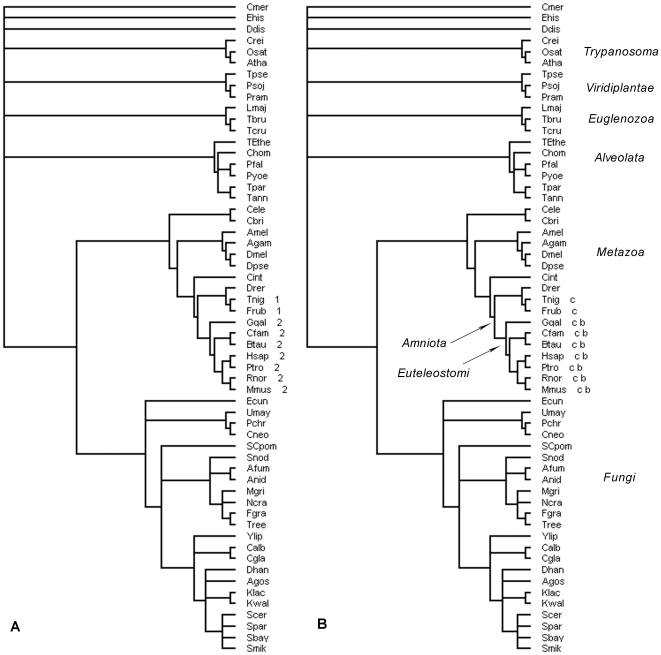
Comparison of domain tree and domain combination tree. Single domains and domain combinations mapped to the eukaryotic tree for SCOP domain a.109.1.1, the Class II MHC-associated invariant chain ectoplasmic trimerization domain. (**A**) The number next to the species name represents the abundance of the domain in the genome of that species. (**B**) The letters represent different combination types. In this case, type b corresponds to N/A∼a.109.1.1 and c represents N/A∼a.109.1.1∼g.28.1.1, where N/A is an unknown domain (no 3D structure, no SCOP id). The complete scientific names of the taxa in this study are listed in the supplementary Table S1.

For instance, Figure 1A shows the domain tree for the Class II MHC-associated invariant chain ectoplasmic trimerization domain (SCOP a.109.1.1), which plays a critical role in the assembly of the major histocompatibility complex (MHC), as well as in MHC II antigen processing [23]. Absent in all bacteria and archaea, this domain appears in the genomes of all *Amniota* except *Danio rerio*. With regard to the principle of maximum parsimony, the evolutionary history of a.109.1.1 can be explicitly derived according to this distribution: a.109.1.1 originated from the root of *Amniota*, and was inherited by all sibling organisms but lost from *Danio rerio*. Note we cannot discount the possibility that the domain exists in *Danio rerio*, since our approach to domain homology detection might not be sensitive enough to detect remote domains. The abundance of domains in the genome of each species allows us to infer possible duplication events as discussed subsequently. In principle, the inference of evolutionary events can be applied to any protein domain and combination thereof.

Using species phylogeny to study the evolutionary origin and history of proteins or protein domains is straightforward and has been widely used [24]–[26]. Investigating the domain architecture of a given protein and deriving its evolutionary origins is a starting point to establish the function of a novel protein. Previous studies have focused on one or a few proteins or domains of interest; in contrast, this work aims to generalize this approach to the whole protein domain repertoire and derive the entire evolutionary history of protein domains. This systematic approach can provide biological insights that can't be achieved by studying individual proteins/domains alone. These insights include the formation of novel domain combinations and their evolution, the divergence of one superfamily into several families, and the general trends in domain evolution.

As discussed, domain combination is a major way of creating new proteins and new functions. Similar to the single domain trees, when and how each domain combination was formed can be observed and mapped to domain combination trees, where each combination type is considered a distinct evolutionary unit. For example, using domain a.109.1.1, there are two combination types that differ by a g.28.1.1 domain, the thyroglobulin type-1 domain (Thyr-1), at the C-terminus of corresponding proteins. The Thyr-1 domain has about 65 amino acids and exists in proteins with various functions and origins; its activity and function is not fully understood [27]. The two combinations are isoforms of the CD74 antigen protein, having a common domain at the N-terminus whose 3D-structure hasn't been solved and thus labeled N/A (unknown for SCOP) in our nomenclature. The domain combination tree of a.109.1.1 (Figure 1B) shows that these two isoforms are evolutionarily related, where isoform I (c in Fig. 1B), N/A∼a.109.1.1∼g.28.1.1, exists in all species that contains a.109.1.1 and is assumed to originate from the common ancestor of *Amniota*, and isoform II (b in Fig. 1B), N/A∼a.109.1.1, first appeared in *Euteleostomi* and thus was most likely created by losing a Thyr-1 domain (g.28.1.1) from the C-terminus after duplication of isoform I.

Not only can the evolution of new combination types be inferred from domain trees, so can the divergence of two evolutionarily related domains. According to SCOP, different domain families within a superfamily (Fold Superfamily, or FSF) originated from a common ancestor, but their sequences have diverged so much that their evolutionary relationship can only be recognized by structural and/or functional relatedness. The distribution of different families within the same superfamily indicates where the divergence event happened in the tree of life.

**Figure 2:**
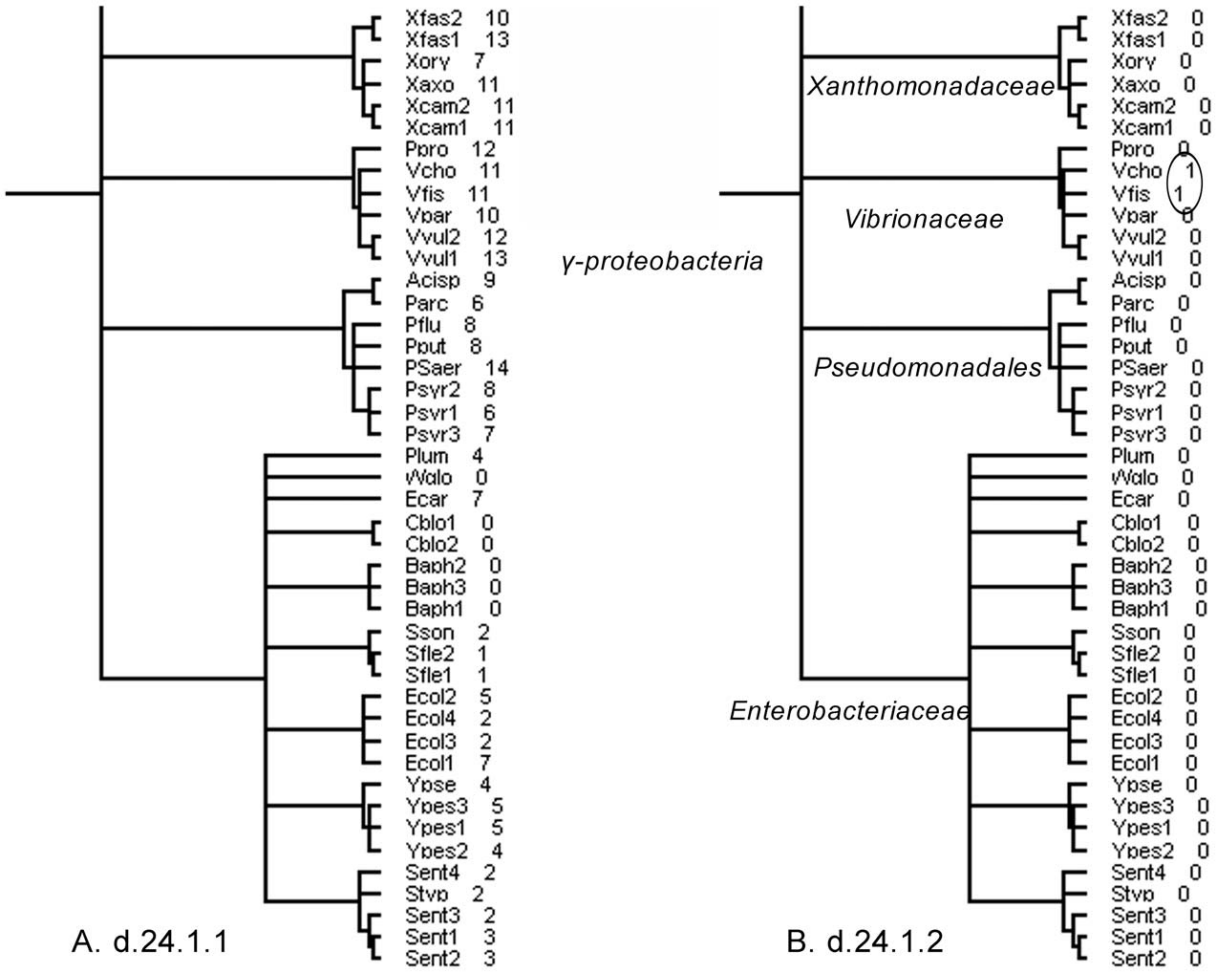
The evolutionary relationship of two families by comparing their domain trees. The domain trees of (**A**) the pilin family (d.24.1.1) and (**B**) the TcpA-like family (d.24.1.2). Both families exist exclusively in bacteria. Only part of the proteobacteria taxa within the bacteria are shown; the complete proteobacteria tree can be found in supplementary Figure S1. The number next to each species represents the abundance of the domain family.

For example, pilin refers to a class of fibrous proteins that oligomerize and form the pilus structure in many bacterial species [28]. Bacterial pili are involved in adhesion to surfaces and conjugate with other bacteria. The pilin superfamily (d.24.1) in SCOP is represented by two families with no detectable sequence similarity, pilin (d.24.1.1) and TcpA-like pilin (d.24.1.2), the latter being the toxin-coregulated pilus discovered in *Vibrio cholera*. As shown in the domain trees of the two families (Figure 2 and Supplementary Figure S1), the pilin family is found in many bacterial species but not in archaea and eukaryotes, so it probably originated in the common ancestor of all bacterial organisms; the TcpA-like pilin family is only found in two species, *Vibrio cholerae* and *Vibrio fischeri*, but not in other bacterial species, so it probably diverged after duplication from the pilin family in one of the ancestral *Vibrio* species. The domain trees explicitly illustrate the evolutionary history of the pilin superfamily (d.24.1), including the origin of the pilin protein family (d.24.1.1) at the root of bacteria, domain duplication in some genomes, domain loss in some bacterial clades, and importantly the divergence of the superfamily and the formation of a new family (d.24.1.2) in the *Vibrio* species.

**Figure 3:**
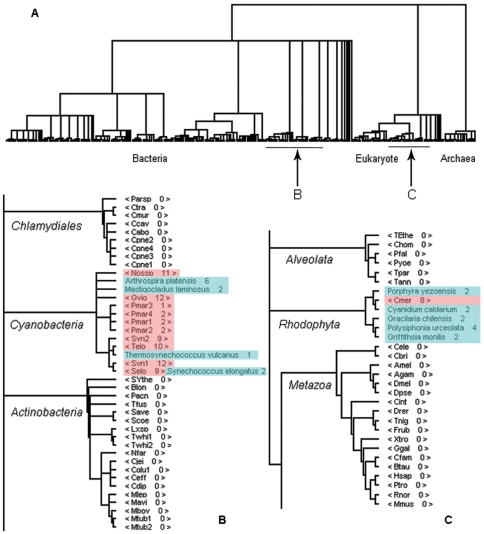
The PDB-validated domain trees of phycocyanin-like phycobilisome proteins (a.1.1.3). (**A**) The patchy distribution of a.1.1.3 on the tree of life. (**B**) Part of the bacteria tree zoomed in; a.1.1.3 exists only in cyanobacteria. (**C**) In the expanded (from Fig. 3A) eukaryote tree, a.1.1.3 only appears in all red algae (Rhodophyta) species, including *Cmer* in our complete genome dataset and five red algae species with solved 3D structures. The red highlight in (B) and (C) indicates domains predicted to exist in the complete genomes based on SUPERFAMILY data; blue highlight in (B) and (C) represents the organisms that comprise the a.1.1.3 domain whose 3D structures are deposited in the PDB.

Not every domain is orderly distributed in the tree of life; some exist sporadically across different clades. For example, the phycocyanin-like phycobilisome proteins (a.1.1.3) are light harvesting antennae of photosystem II [29]. The domain tree of a.1.1.3 (Figure 3A) shows that it only exists in two evolutionarily distinct phylogenetic groups, cyanobacteria in the bacterial superkingdom (Figure 3B) and red algae in eukaryotes (Figure 3C). The sporadic distribution most likely results from horizontal gene transfer and strongly supports the endosymbiosis theory; the acquisition of the photosynthesis system in red algae from endosymbiotic cyanobacteria.

In summary, protein domains mapped to species trees illustrate evolutionary processes such as the origin of domains, domain loss and transfer, domain combination and divergence. In principle, the entire evolutionary history of every domain can be visualized and derived based on the phylogenetic distribution of that domain. Subsequently domain combinations can be mapped with a complexity that is related to the number of combinations of a given domain; some domains are promiscuous and appear in many families and superfamilies, others do not.

### General Trends in Protein Domain Evolution

Mapping of domains and domain combinations to species trees is too time-consuming to do manually. Our approach (see methods), similar to the approach introduced by Snel et al. [30], aims to predict the presence or absence of protein domains in ancestor organisms based on their distribution in present day organisms. Four evolutionary processes govern the presence or absence of a domain at each node in the tree: vertical inheritance, domain loss, horizontal gene transfer (HGT) and domain genesis. (Domain duplication and recombination do not affect domain presence.) Each process is assigned an empirical score according to their estimated relative probability of occurring during evolution, and the minimum overall score depicts the most parsimonious evolutionary processes of each domain or combination (see methods).

Table 1 lists the predicted number of domains and domain combinations originated in the major lineages of the tree of life. 1984 domains (at the family level) are predicted to be in the root of the tree (with the ratio R_hgt_ = 12), accounting for more than half of the total domains (3464 families in SCOP 1.73). This prediction is significantly higher than what is generally believed [5], [31], [32]. There are several reasons to account for the discrepancy. First, previous attempts focused on universal and ubiquitous proteins (or domains) in LUCA [5], so one protein has to exist in the majority of species in each of the three superkingdoms (usually 70%–90%) to be considered as LUCA protein [32]. Second, the root of the tree is still not solved. Thus any domains that are shared by two superkingdoms are counted as originating in the LUCA. Endosymbiosis of mitochondria and chloroplasts and horizontal gene transfer across superkingdoms can result in the same effect, which is moving the origin of protein domains towards the root. Third is our limited knowledge of protein domains. On average nearly 40% of predicted ORFs in the genomes under study cannot be assigned to any known domain. When assigned in the future they may turn out to be species or lineage specific domains that emerged relatively late on the tree of life. There are also a significant number of domains which emerge at the root of bacteria and eukaryotes. Likewise, this can be explained by the unresolved early evolution at the origin of bacteria and eukaryotes. Indeed, with regards to the species in our dataset, the bacteria tree contains 18 kingdoms and the eukaryote tree contains 11.

**Table 1:** Origin of protein domains and domain combinations along the tree of life.

Taxa	# Dm	# Cb	# Cb/Dm	Avrg Length
cellular organisms	1984	4631	2.33	2.15
Archaea	13	104	8.00	2.71
Euryarchaeota	14	176	12.57	2.89
Thermoprotei	4	43	10.75	3.02
Bacteria	144	1066	7.40	2.81
Actinobacteria	19	544	28.63	3.21
Bacteroidetes	2	73	36.50	3.68
Chlorobiaceae	2	53	26.50	3.19
Chlamydiales	2	17	8.50	3.00
Chloroflexi	3	60	20.00	3.60
Cyanobacteria	43	303	7.05	3.16
Mycoplasma	4	35	8.75	2.69
Thermoprotei	4	43	10.75	3.02
Firmicutes	28	173	6.18	3.03
Clostridia	3	112	37.33	3.19
Bacilli	7	50	7.14	2.74
Bacillales	9	62	6.89	2.89
Lactobacillales	3	35	11.67	3.00
Mycoplasma	4	35	8.75	2.69
Thermoprotei	4	43	10.75	3.02
Proteobacteria	87	773	8.89	3.01
Alphaproteobacteria	8	163	20.38	3.03
Rhizobiales	6	195	32.50	3.52
Rickettsia	2	21	10.50	2.81
Betaproteobacteria	2	54	27.00	3.35
Burkholderia	15	155	10.33	3.63
Epsilonproteobacteria	1	22		3.23
Deltaproteobacteria	11	196	17.82	3.27
Gammaproteobacteria	22	145	6.59	3.01
Enterobacteriaceae	32	109	3.41	2.85
Eukaryota	492	6056	12.31	3.10
Alveolata		58		3.91
Trypanosomatidae	3	120	40.00	3.76
stramenopiles		58		4.03
Viridiplantae	9	464	51.56	3.35
Chlorophyta	1	116		3.61
Streptophyta	15	320	21.33	3.70

Taxa	# Dm	# Cb	# Cb/Dm	Avrg Length
Fungi/Metazoa group	37	875	23.65	3.57
Fungi	10	286	28.60	4.09
Dikarya	14	473	33.79	3.89
Basidiomycota	2	89	44.50	4.37
Ascomycota	3	215	71.67	4.33
Saccharomycetales	2	66	33.00	4.08
Pezizomycotina	15	840	56.00	4.27
Eurotiomycetidae	1	96		4.38
Sordariomycetes	2	149	74.50	4.57
Metazoa	58	557	9.60	4.00
Eumetazoa	47	841	17.89	3.86
Bilateria	17	1306	76.82	3.98
Chromadorea	3	125	41.67	4.20
Coelomata	20	1968	98.40	4.75
Protostomia	1	158		5.18
Pancrustacea	6	85	14.17	5.05
Deuterostomia	5	254	50.80	5.00
Chordata	15	754	50.27	5.29
Euteleostomi	19	1767	93.00	5.85
Clupeocephala		118		7.30
Tetrapoda	2	322	161.00	5.90
Amniota	3	252	84.00	6.75
Mammalia	2	297	148.50	5.66
Mammalia	2	297	148.50	5.66
Theria		347		7.06
Eutheria	5	1773	354.60	5.74
Laurasiatheria	2	150	75.00	6.05
Afrotheria		22		5.27
Euarchontoglires		411		6.18
Glires		53		5.43
Primates		61		6.51
Haplorrhini		19		6.37
Catarrhini	1	173		6.65
Hominidae	1	79		9.73
Homininae		115		10.17

The total number of domains and domain combinations is 3464 and 116,400, respectively. Columns from left to right list the number of domains and combinations originated from each major lineage, the ratio of the number of combinations over that of domains, and the average number of domains per protein rooted at each lineage. Taxa are arranged according to their phylogenetic classification. Some nodes could not be evaluated since they derive from 0 or 1 domains.

**Figure 4:**
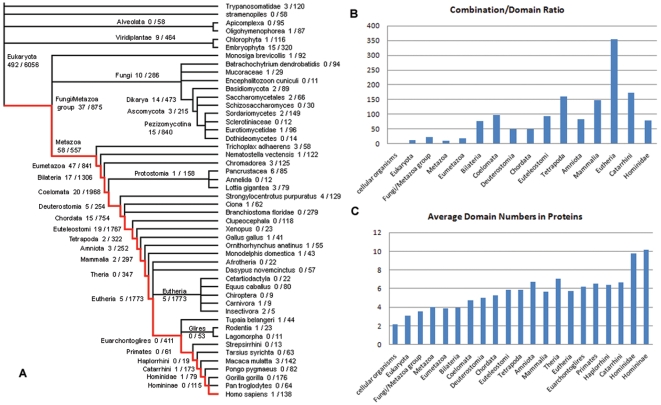
The general evolutionary trend of protein domains and domain combinations. (**A**) The predicted number of domains/domain combinations originating at each node on the eukaryotic tree. (**B**) The combination/domain ratio at each node along the evolutionary path from the root of the tree to *Homo sapiens* indicated by the red line in Fig. 4A. (**C**) The average number of domains in the domain combination originating from each node along the same evolutionary path.

Notwithstanding, these data suggest that a large proportion of protein domains were invented in the root or after the separation of the three major superkingdoms but before the further differentiation of each lineage. When tracing outward along the tree from the root, the number of novel domains invented at each node decreases (Figure 4A). Many branches, and hence species, apparently do not invent any domains. As previously discussed, this might be a result of the incomplete knowledge of lineage specific domains. Given the data we have it is estimated that during the approximately two billion years after the appearance of the first eukaryotic cell, only 831 domains, less than 1/4 of the total number of domains, has been invented.

Conversely, the evolution of domain combinations shows the opposite trend. There are 4631 combinations at the root of tree, which accounts for only 4% of total combinations. Relatively more combinations were invented in the descendant nodes of the tree, as indicated by the increase in the ratio of number of new combinations and domains at each node along the tree of life (Figure 4B), and the relative increase in combinations is more significant in eukaryotes than in prokaryotes (Table 1). This combined evolution of domains, and combinations thereof, suggests that once protein domains have been generated and inherited in genomes, biological organisms tend to create new proteins and functions through duplication and recombination of existing domains, rather than create new domains *de novo*, in accordance with the general trend of genome evolution by means of duplication and recombination [33].

Given the origin of every domain combination, we can determine the average number of domains in proteins originating at each node in the tree of life (Figure 4C). As shown in Table 1, there is a general trend of increasing domain numbers per protein during evolution, but at different degrees in the three superkingdoms. The number increases from 2.15 for proteins originating in LUCA, to more than 6 in higher vertebrates, but only increasing to about 3 in contemporary bacteria and archaea. This observation confirms previous findings [2] on the differences in domain numbers per protein in prokaryotes and eukaryotes. In addition, the difference does not result from more ancestral short proteins being inherited by prokaryotes, because even novel proteins invented later in evolution by prokaryotic lineages are much shorter than those invented by eukaryotes.

## Discussion

### Phylogeny and Taxonomy

The major problem with the representation of a taxonomy-based phylogeny is that it is not a well-resolved tree that reflects every bifurcation and speciation event. The six or seven major hierarchical levels of the taxonomy result in multiple clades at the same level whose evolutionary relationships are not determined. As a result, the evolutionary origin of a domain or domain combination determined by the taxonomy-based phylogeny is biased towards the higher levels of the phylogeny. This bias in evolutionary origin also results in an over-estimation of the extent of gene loss. For instance, a domain exists in archaea and eukaryotes but not in bacteria. Because the NCBI taxonomy does not have the branching order for the three superkingdoms, according to our method, the origin of this domain is in LUCA and it was lost in the bacterial branch. If, we suppose, bacteria diverged first from the root, followed by the branching of archaea and eukaryotes, then the derived origin of the domain is located in the common ancestor of archaea and eukaryotes, and the bacteria never contained this domain.

The problem brought about by taxonomy can be corrected by using bifurcating phylogenetic trees that contain detailed evolutionary relationships for every taxon. Currently, many branches of the tree of life are still unsolved and in debate, such as the separation of the three superkingdoms and the divergence of bacteria and eukaryote taxa. In those cases, the taxonomy-based phylogeny that allows multiple leaves under a node must be used. As the phylogenetic tree of life becomes more accurate and reliable, our understanding of the evolutionary history of protein domains will also improve.

### Genome Coverage of Domain Assignments

The average domain coverage of each genome is between 40% and 60%; genes in the rest of the genomes are either unannotated or lack a 3D structure, and are in many cases species-specific genes. New folds and superfamilies are assigned to protein structure classification schemes as more protein 3D structures are solved; this increased the average domain coverage of genomes from 53% in SCOP 1.63 (765 folds, 1232 FSFs) to 60% in SCOP 1.73 (1086 folds, 1777 FSFs) over a period of four years (Supplementary Text S2). The use of sequence-based protein domain classifications, such as Pfam [34], increases the coverage of domain assignments, but looses remote evolutionary linkages only defined by structural conservation. Therefore, although domain coverage will continue to increase as structural data accumulate, we anticipate that this will plateau and we cannot expect complete coverage in the near future.

Nevertheless, the current genome-wide domain assignment data are copious and significant enough to make evolutionary arguments, such as reconstructing species phylogenies based on protein domain content [5]. In this study, the evolutionary histories of known domains are not affected, but many domain combinations include unassigned parts that determine the identity of each domain combination and require further analysis. Unassigned protein regions have been discussed before, but no satisfactory method to deal with the problem exists [35]–[37]. In this work, we choose a simple approach by treating an unassigned region in a protein as a new domain if it has a significant length (>50). This method sets a lower boundary for depicting the existence of unknown domains, but sets no limit on the number and type of unknown domains in one unassigned segment. As a result, this method groups multiple non-identical domain combinations as one (Supplementary Text S2), which reduces the total number of types of combinations, moves the predicted evolutionary origin of each domain combination towards the root of the tree, and in some case increases the number of independent genesis events of domain combinations. Given these artifacts, the identity and evolution of individual domain combinations needs careful consideration, but the general trend in the evolution of domain combinations with respect to protein domains still holds.

### Evaluation of Genesis/HGT to Loss Ratio

**Figure 5:**
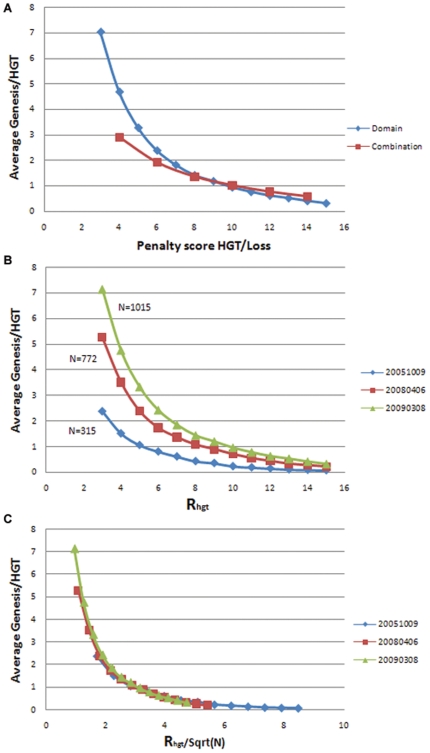
The predicted average HGT or independent genesis events. (**A**) The average number of HGT or independent genesis per domain/combination with respect to the relative penalty score of Genesis/HGT to loss varying from 3 to 15. (**B**) Comparison of average Genesis/HGT vs. Rhgt for three SUPERFAMILY releases: Oct 9^th^ 2005, Aug 6^th^ 2008 and Mar 8^th^ 2009, containing 315, 772 and 1015 species respectively. (**C**) The same plot with the ratio normalized by an empirical factor. The new ratio is R_n_ = R_hgt_/Sqrt(N), N is the total number of species in each release.

As shown in the methods section, the genesis/HGT to loss ratio R_hgt_ is the major factor of our method in determining the evolution of domains. In this section, its value and the implication to our predictions and conclusions are discussed. Increasing this ratio indicates it is more difficult for HGT or independent genesis to happen compared to domain loss, lowering this ratio allows more HGT or independent genesis events in deriving the evolutionary origin and history of each domain or combination. Therefore, the average number of HGT or genesis event happened in the history of every domain will decrease monotonically with the changing of the ratio R_hgt_ (Figure 5A). When the ratio is 4, every domain and domain combination has HGT or independent genesis events in their history 4.7 and 2.9 times on average, respectively. As R_hgt_ increases, the number of domain genesis events falls to 0, which means every domain/combination was only invented once in history with no HGT and no convergent evolution. The slope of the curve is very inclined when the ratio is small (R_hgt_<8), implying that changing the value of the score will have significant impact on the history of domains; whereas the slope becomes flatter with larger ratios.

As indicated in Figure 5A, domain and domain combination undergo different HGT or independent genesis events given the same R_hgt_ ratio. Domain combinations show more genesis or HGT than single protein domains in the flat region (R_hgt_>9) of the graph. One explanation is that domain combinations are relatively easy to reinvent from existing domains multiple times in different lineages during evolution. It is also possible that, as explained in the later section, because protein domains are less sensitive to HGT than proteins or domain combinations, some HGTs among closely related species are enclosed in the same evolutionary profile and not revealed.

The penalty score for genesis/HGT is an empirical score based on statistical analysis, its true value must be evaluated from the accuracy of its predictions [30]. In addition, it is also related to the number of species and the topology of the species tree. As one can imagine, the ratio of genesis/HGT vs. loss is a cutoff for determining if the evolutionary distance of two given species with respect to the given phylogenetic tree is large enough that a genesis/HGT events between the two species is evolutionarily more favorable than multiple losses among all other progenies of the common ancestor of the two species. The distance of any given species within the tree will increase as the total number of species increases and the tree topology changes. Figure 5B shows the comparison of three releases of the SUPERFAMILY dataset, Oct 9^th^ 2005, Apr 6^th^ 2008 and Mar 8^th^ 2009, with a total number of completed sequenced species of 315, 772 and 1015, respectively. With the increase in the total number of species, the average genesis/HGT also increases under the same penalty score. To normalize this effect, an empirical factor, the square root of the total number of species (N), is used such that Rn = R_hgt_/sqrt(N). As shown in Figure 5C, the three curves converge, which indicates that the relationship between average genesis/HGT and the ratio is independent of the total number of species studied.

**Figure 6:**
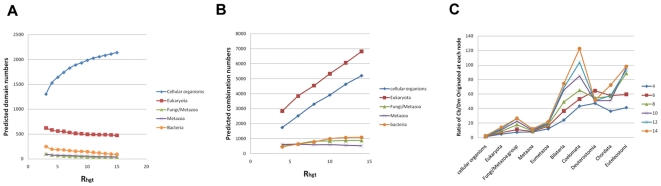
The impact of the relative penalty score R_hgt_. (**A–B**) The predicted numbers of domains (**A**) and domain combinations (**B**) originating from six ancestral nodes (LUCA, Eukaryota, Fungi/Metazoa, Metazoa and Bacteria) with respect to different R_hgt_ values (**C**) The impact of the R_hgt_ value on the ratio of the number of combinations over the number of domains originated at each ancestral node along the same evolutionary path as in Figure 4B.

The predicted numbers of domains and domain combinations originated at each node in the tree is also determined by the changes in R_hgt_. Figure 6A and 6B lists the predictions at five ancestor nodes (Cellular Organism, Eukaryota, Bacteria, Fungi/Metazoa and Metazoa) under different R_hgt_ values (complete data is provided in supplementary Table S2). For protein domains (Figure 6A), as R_hgt_ increases, only domains originated in LUCA increases; the value increases from 1303 at R_hgt_ = 3 to 2140 at R_hgt_ = 15, as can be expected that the increase of the penalty for HGT and genesis will lead to more loss and more at the ancient root. This indicates that even with a very low R_hgt_ ratio (which is very unlikely because on average each domain undergoes 7 HGT events when R_hgt_ = 3, Figure 5A), a significant number of domains were invented before LUCA, and the general conclusion is not affected by the changes of R_hgt_ value. In the case of domain combinations (Figure 6B), besides LUCA, other ancient nodes contains more novel combinations as the penalty score goes up. Since the impact of the R_hgt_ value is identical for domains and combinations, differences arise because more combinations were invented late in evolution (Figure 4). For those that only exist in eukaryotes, the increase in the R_hgt_ value will push the predicted root towards the root, up till the common ancestor of eukaryote. The differences in the two cases, however, does not affect our previous conclusion that during evolution novel functions are invented by means of new combinations rather than novel protein domains. As shown in Figure 6C, under different R_hgt_ scores, the increase of the ratio Cb/Dm still holds, and it tends to be higher at greater R_hgt_ values.

Most importantly, although the penalty score R_hgt_ affects our calculation and hence the prediction of the evolution of protein domains and combinations, it does not change our main conclusion concerning the general trend of protein domain evolution. The proper value of R_hgt_ has a lower bound, which can be denoted by excess HGT or genesis events per domain, and an upper bound, which is determined by the number of species and the overall tree structure. As a statistic score, the value of R_hgt_ can only be derived empirically; we use a value of 12, located in the flat region in our analysis.

### Horizontal Gene Transfer

Horizontal gene transfer (HGT) is a major force in the evolution of prokaryotes. Genome comparison suggests that up to 20–30% of genome variation is due to this process [38]. Given that HGT is so pervasive some have claimed that the reconstruction of a universal tree of life is not possible [39]. Our approach assumes the existence of a discrete species phylogeny that represents the entire history of life and thus HGT is a critical process that must be considered.

The methodology used here, namely the use of multiple sequence alignments to construct hidden Markov models (HMMs), means that the HMM does not distinguish orthologs and paralogs. In other words, a protein domain represents an ensemble of evolutionarily-related sequences that include both orthologs and paralogs. Moreover, after domain assignment only the presence and absence of domains are evaluated in domain trees and domain combination trees. Thus HGT of homologous proteins within closely related species (within-phylum HGT), which is estimated to happen extensively and more vigorously than HGT between phyla [40], is indistinguishable from vertical inheritance and will not be found by our approach. Only foreign non-homologous proteins that are transferred from distantly-related species (HGT between phyla) and significant enough to give rise to a patchy domain distribution across the evolutionary tree can be recognized by our domain trees. In summary, while viewing phylogeny based on individual genes or proteins might be complicated by massive HGT, phylogeny viewed by protein domains are expected to be more robust and tolerant to HGT and protein domain trees can often reveal substantial HGTs when they occurred (e.g., Figure 3).

### A Domain Centric View of Evolution

Typically the first step in understanding the properties and function of a protein is to analyze its domain architecture. Many domains have different functions in different proteins, especially when in combination with other dissimilar domains. The specific function of each domain and their domain combinations are better understood when considered in conjunction with their evolutionary relationships. Previous studies of protein evolution emphasized finding homologs to the full-length protein, thus neglecting evolutionarily related proteins that differ by one or more domains. Our domain-level approach investigates the evolution of each individual domain and domain-based evolutionary processes, such as domain fusion and fission, which give rise to various domain combinations. The methodology is relatively straightforward and domains and domain combinations can be updated as new genomes are sequenced. Moreover, as more protein structures are determined and more domains assigned the repertoire of domains that can be mapped to a given genome will also increase.

## Methods

### Data Source

The same procedure as discussed in our previous work [5] is used to make domain assignments, but using more recent data. SCOP 1.73, released in Sep 2007, classifies protein domain into 1086 folds, 1777 superfamilies and 3464 domain families; SUPERFAMILY release 2009-03-08 includes complete genome sequences for 54 archaea, 732 bacteria and 229 eukaryotes (a total of 1015 species). In this release of SUPERFAMILY not only did the number of complete genomes increase, but domain assignments have been calculated at the family level, which allows us to study the divergence of a superfamily into families. We use the same e-value cutoff (1e-4) as previously when analyzing these data.

The NCBI taxonomy used here was retrieved on March 8th, 2009 from the NCBI Entrez Database [41]. Of the approximately 300,000 species included in this taxonomy, 1490 species were used here, including the 1015 species with complete genomes and their ancestor species tracing back to the root of the tree.

### Domain Combination

The domain assignment provided by the SUPERFAMILY database gives the position and length of each domain within a given protein. Thus for every protein, its domain composition and domain order relative to the protein sequence is readily available. However, unassigned parts of the protein sequence introduce complications in designating domain combinations. Using the SCOP definition, domains are identified which cover between 40–60% of complete genomes. Among the 6.2 million open reading frames (ORFs) from all 1015 complete genomes used in this study, 38.9% of the ORFs are fully assigned, a further 22.4% of the ORFs have partial domain coverage and 38.7% of the ORFs have no coverage at all. Unassigned regions could be linkers between domains, one domain, or even multiple domains. Unassigned regions make the identification of each combination type difficult; one solution is to only consider fully annotated proteins, but this excludes most combinations.

In this work, we consider each unassigned region as a potential domain and include it as part of our combination nomenclature. An unassigned region is considered as one unknown domain if it is longer than 50 amino acids. (This simplification ignores the cases of multiple domains in the unassigned region, the implication of which are discussed in the supplementary Text S2) Overall, in the current analysis there are approximately 116,400 types of domain combination, with 20,397 types accounting for 95% of all combinations. Many combinations are species specific or exist in a limited number of organisms. Conversely, some combinations, which originated in the last universal common ancestor and duplicated multiple times during evolution, are very abundant. In general, the abundance of domain combinations follows a power law [42].

### Domain and Domain Combination Tree Construction

We translated the NCBI taxonomy plain text files into a standard tree file format (See Supplementary material Dataset S1). A domain tree is then constructed by labeling and characterizing each leaf organism of the phylogenetic tree by the type and number of protein domains in its genome. This tree construction method is not limited to species with complete genomes; any protein sequence from other species can be incorporated. For instance, 3D structures from the Protein Data Bank (PDB) [43] were extracted from various organisms and their positioning on the tree can be used to validate the predicted domain organization found using domain or combination trees (Figure 3).

### Prediction of the Origin of a Domain

The origin of a domain can be found by tracing back the existence of domains on the tree of life based on the principle of parsimony. Four evolutionary processes, vertical inheritance, domain loss, horizontal gene transfer (HGT) and domain genesis can change the status of domain content. We assign each process a penalty score according to their relative likelihood of occurring during evolution. Vertical inheritance is the default evolutionary process and its penalty score is 0. The relative penalty score for gene loss is assigned as 1. Domain genesis indicates the origins of protein domains. Although convergent evolution exists, a recent study indicated that domain convergence and multiple domain genesis are rare and most domains emerged only once during evolution [11]. Horizontal Gene Transfer (HGT) is also rare when compared to domain loss. Moreover, HGT or multiple domain genesis events can give rise to the same apparent domain distribution patterns on the tree of life and hence are not distinguishable (see supplementary Text S1). Therefore, the penalty score for either domain genesis or HGT is defined as R_hgt_ (R_hgt_>1), indicating the relative likelihood of domain genesis or HGT with respect to loss. As the only parameter in this model, the genesis or HGT to loss ratio R_hgt_ largely influences the outcome of the prediction, so it is evaluated at different values.

To find the ancestor domain content that best fits the current domain distribution is equivalent to finding the most parsimonious present/absent dataset for each node on the tree so as to minimize the total score for the whole tree. The details of the iterative algorithm we developed can be found in the supplementary Text S1. The origin and evolution of domain combinations are derived using the same procedure as that for single domains. Although the processes of invention, loss and transfer of domain combinations are different from single domains, if each domain combination is considered as individual evolutionary unit, the above analysis still holds. The evolutionary difference for single domains and combinations is incorporated into the relative ratio R_hgt_.

## Supporting Information

Text S1 Algorithm developed to derive the history of protein domains
(0.07 MB DOC)

Text S2 Discusses some issues related to genome coverage and unassigned regions.
(0.09 MB DOC)

Figure S1 Complete proteobacteria domain tree of the pilin family and TcpA-like family
(1.19 MB TIF)

Table S1 Species with complete genomes involved in this study
(0.81 MB DOC)

Table S2 Predicted number of protein domains originated from each ancestor node in the tree of life with respect to different R_hgt_ values.
(0.49 MB DOC)

Dataset S1 NCBI phylogenetic tree involved in this study
(0.02 MB TXT)
