# Supplementary material for: The Evolutionary History of Protein Domains Viewed by Species Phylogeny
Source: PLoS One. 2009 Dec 21;4(12):e8378. doi: 10.1371/journal.pone.0008378 (PMC2794708; doi:10.1371/journal.pone.0008378)
Supplement: Text S1 — Algorithm developed to derive the history of protein domains (0.07 MB DOC) [file pone.0008378.s001.doc]

Supplementary Material Text S1

Method

Prediction of the origin and evolutionary history of protein domains

## Method

The question is, given a phylogenetic tree and the distribution of a domain or domain combination among all the leaf species, how can we find its evolutionary origin and the present/absent status of the domain (combination) in all the ancestor species in the tree? That is, we want to reconstruct the protein domain content of ancestor organisms in the tree of life. This approach will explicitly show which domains were present in the ancestral genomes and how the domain content of ancestral and present day genomes has been shaped by the processes of domain loss, domain duplication, HGT, and domain genesis.

Assume we have a phylogenetic tree and the present day presence pattern of a certain domain, as hypothetically illustrated in Figure S2A. Here, we only consider the presence and absence of domains, which are represented as 1 and 0, respectively. There are four evolutionary processes that influence the present state of domain content, namely, vertical inheritance, domain loss, horizontal gene transfer (HGT) and domain genesis. (Domain duplication and recombination do not affect domain content.) We empirically assign each process a penalty score according to their relative ease of occurrence during evolution (Table S3). See text for a description for how these values were estimated.

| Vertical Inheritance | 0 |
| --- | --- |
| Domain Loss | 1 |
| HGT | 12 |
| Domain Genesis | 12 |

Table S3 Penalty scores for evolutionary processes.

The score for vertical inheritance is set to be 0, because it is the default process that ensures carrying of genetic information from parents to descendants. HGTs are considered less likely than domain loss, so their penalty score is set higher. Assuming no convergent evolution, domain genesis, the *de novo* creation of domains, took place only once for each domain during evolution. HGT has the effect of causing multiple origins for a domain. If a domain trees shows N origins, one belongs to domain genesis, the other N-1 are the result of HGT. Since both domain genesis and HGT result in one origin in the tree, the penalty score for HGT and domain genesis are set the same.

A. Domain content in current species

0

0

0

0

0

1

1

0

0

1

1

1

0

0

B. Prediction of ancestor states without HGT

1

1

0

1

0

1

1

1

1

0

0

0

0

0

1

1

0

1

1

1

0

1

1

1

0

0

1

C. Prediction of ancestor states with HGT

0

0

0

1

0

1

0

0

0

0

0

0

0

0

1

1

0

1

1

1

0

1

1

1

0

0

0

Figure S2. Two possible evolutionary histories for domains (B and C) that result in the same current domain distribution (A)

To find the ancestor domain content that best fits the current domain distribution is equivalent to finding the most parsimonious present/absent dataset for each node on the tree, in order to minimize the total score for the whole tree.

0

0

1

1

1

0

0

1

Inherit

Loss

Genesis / Transfer

0

1

R_hgt_

Figure S3. The four possible evolutionary events between parent and child

Each node has two possible conditions, presence or absence. There are four possible combinations between parent and child, as illustrated in Figure S3. The most straightforward method to find the most parsimony dataset would be to traverse the whole tree and test every possible combination. However, the calculation complexity is 2^N^, N being the total number of nodes on the tree. The exponential complexity of this algorithm is not applicable when the number of species in the tree is large. Rather, we developed an iterative algorithm that at each step uses the relative penalty score of loss-to-transfer as a cutoff to simplify the calculation.

For each internal node C on the tree, define S_node_(0) and S_node_(1) as the minimum score of the clade defined by the root node C, assigning N as 0 or 1, the two possible states of C, respectively. S_node_ for each node is evaluated according the scores of sibling nodes, specifically:

$\begin{matrix} S_{node}(0)=\sum_{children} \min\left[\left(S_{pair}(0,0)+S_{child}(0)\right),\left(S_{pair}(0,1)+S_{child}(1)\right)\right] \\ S_{node}(1)=\sum_{children} \min\left[\left(S_{pair}(1,0)+S_{child}(0)\right),\left(S_{pair}(1,1)+S_{child}(1)\right)\right] \end{matrix}$ (1)

where, S_pair_(parent, child) is defined as the inheritance score between parents and children, whose values are S_pair_(0,0) = S_pair_ (1,1) = 0, S_pair_(1,0) = 1 and S_pair_(0,1) = R_hgt_, as shown in Figure S3.

Since the presence/absence states of both parent and children are to be determined, the above equations consider every possible combination and evaluate the minimum score for each sibling node. In both equations the first terms represent the case when the state of the sibling node is 0; the additions of S_pair_ account for the inheritance score between parent and children. The second terms represent the score assuming the state of the sibling node is 1. The minimum score of the two cases is the contribution of each sibling node to the score of the whole clade.

The minimum score of each node can be derived by the iterative process, till the leaf nodes, whose states are given and S_leaf_ is 0 for all leaves. For any given R_hgt_, the minimum penalty scores for each node only need to be calculated once, so the computational complexity is linear for this algorithm.

In this algorithm, HGT is not considered explicitly, because a HGT event is mathematically equivalent to two independent domain genesis events (Figure S2-C). To some degree, these two events (HGT and domain genesis) are not discernable given only the domain distribution on the tree. If the lowest score involves two or more domain genesis ( 0->1 transition ) events in different sibling branches of this node, it indicates multiple domain genesis or HGT events took place between sibling branches. The outcome of this process is depicted pictorially in Fig S2.
