# Supplementary material for: The Evolutionary History of Protein Domains Viewed by Species Phylogeny
Source: PLoS One. 2009 Dec 21;4(12):e8378. doi: 10.1371/journal.pone.0008378 (PMC2794708; doi:10.1371/journal.pone.0008378)
Supplement: Text S2 — Discusses some issues related to genome coverage and unassigned regions. (0.09 MB DOC) [file pone.0008378.s002.doc]

Supplementary Material Text S2

Discussion:

- Genome coverage by protein domains increase gradually
- Analysis the impact of unassigned region

## Genome coverage

| SCOP | 1.65 | 1.69 | 1.73 |
| --- | --- | --- | --- |
| Escherichia coli K12 | 56% | 60% | 64% |
| Caulobacter crescentus CB15 | 56% | 57% | 62% |
| Bacillus subtilis ssp. subtilis 168 | 54% | 59% | 62% |
| Mycoplasma genitalium G-37 | 52% | 55% | 57% |
| Synechocystis sp. PCC 6803 | 53% | 54% | 60% |
| Deinococcus radiodurans R1 | 54% | 56% | 60% |
| Halobacterium sp. NRC-1 | 53% | 56% | 59% |
| Average | 54.2% | 56.8% | 60.8% |
|  |  |  |  |
| SCOP | 1.65 | 1.69 | 1.73 |
| Fold | 800 | 945 | 1086 |
| Superfamily | 1294 | 1539 | 1777 |
| Family | 2327 | 2845 | 3464 |

**Table S4. For selected species, the percentage of the genome covered by protein domains as defined by different version of SCOP.**


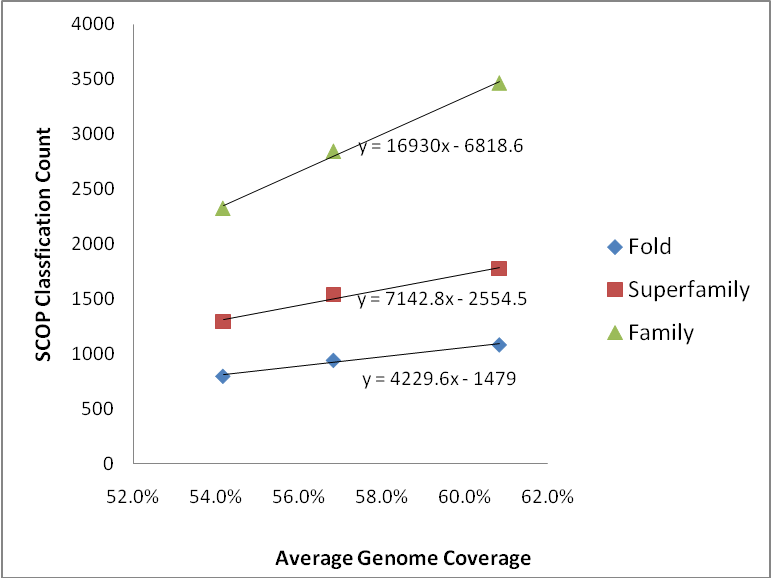


**Figure S4. The increase in average domain coverage in complete genomes with respect to the growth of the SCOP structure classification.** A simple linear extrapolation indicates that, with 100% genome coverage, the SCOP classification will contain approximately 2800 Folds, 4600 Superfamilies and 10000 Families.

## Unassigned Regions

Figure S5 is a hypothetical example to show the influence of unassigned regions on the evolution of domain combinations. Fig S5A depicts the distribution of four types of combination associated with domain A (AB, AC, ACD and AE) found in current organisms and the predicted evolutionary origin of each combination in the tree. If among these domains only domain A is known, B, C, D, E are unassigned region, according to our approach, all four types of combination are represented by A− ( ‘−‘ indicates unassigned region), so the four combinations are grouped into one type (Fig S5-B). Meanwhile, the evolutionary origin of A− tends to move toward the root of the tree, and multiple combination geneses (convergent evolution) may occur. Even with these artifacts and the inevitable incompleteness of domain coverage, this approach still allows us to approximate the evolution of domain combinations. The total number of protein domain combination is actually much more, and our conclusion about the general trend of the evolution of domains and combinations still holds.

**Figure S5. An example of the evolution of domain combination with unassigned regions.** (A), the true combination tree; (B) the predicted combination tree with unassigned regions.

(A)

AE

AC

AC

AB

AC

ACD

AE

AE

AB

AB

A−

A−

A−

A−

A−

A−

A−

A−

(B)
