# Supplementary figures and images for: The Evolutionary History of Protein Domains Viewed by Species Phylogeny
Source: PLoS One. 2009 Dec 21;4(12):e8378. doi: 10.1371/journal.pone.0008378 (PMC2794708; doi:10.1371/journal.pone.0008378)

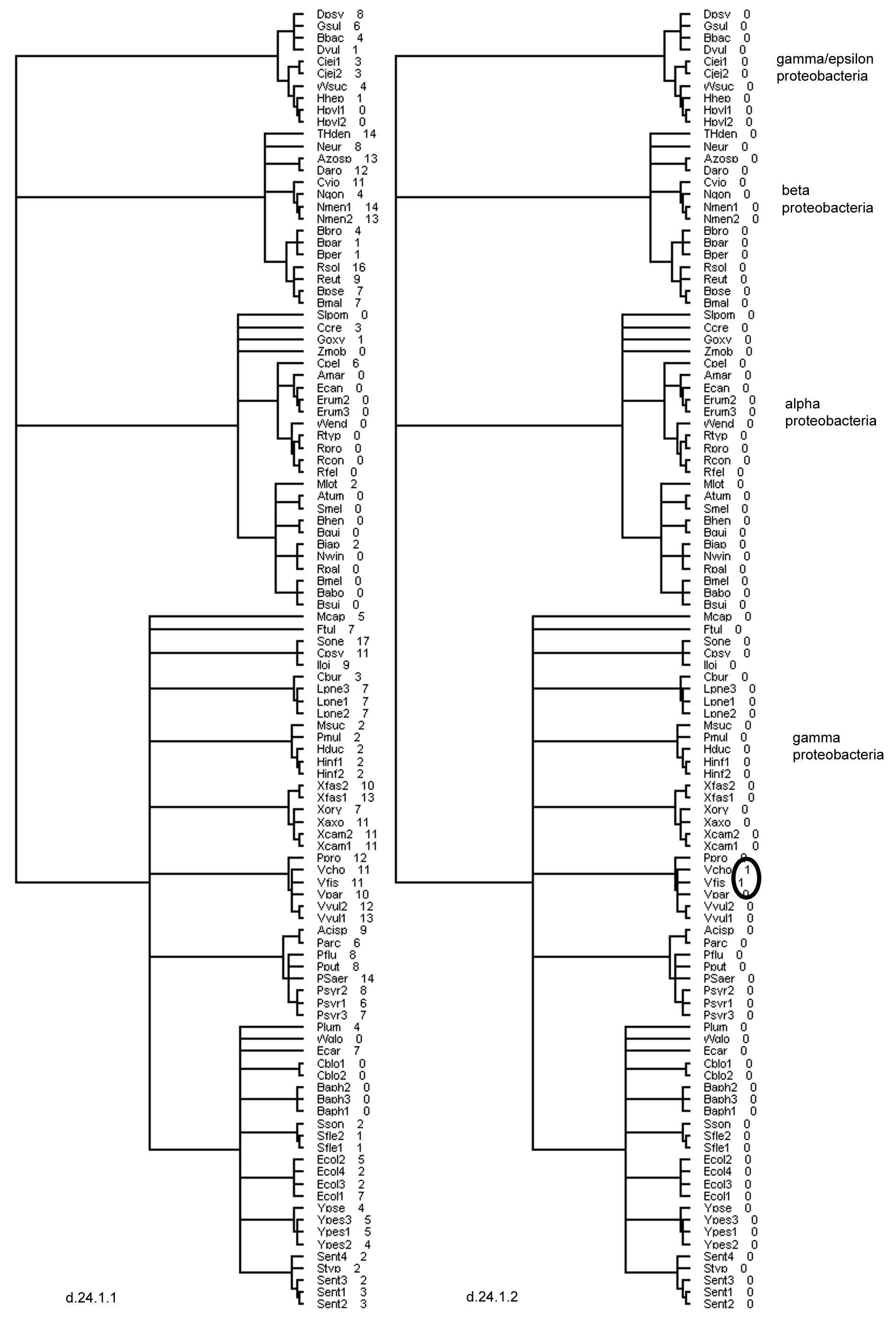

Supplement: Figure S1 — Complete proteobacteria domain tree of the pilin family and TcpA-like family (1.19 MB TIF) [file pone.0008378.s003.tif]
