# Supplementary material for: The Evolutionary History of Protein Domains Viewed by Species Phylogeny
Source: PLoS One. 2009 Dec 21;4(12):e8378. doi: 10.1371/journal.pone.0008378 (PMC2794708; doi:10.1371/journal.pone.0008378)
Supplement: Table S1 — Species with complete genomes involved in this study (0.81 MB DOC) [file pone.0008378.s004.doc]

Table S1-1 Eukaryote species list

| Abbreviation | Organism |
| --- | --- |
| Apis | Acyrthosiphon pisum |
| Aaeg | Aedes aegypti 49_1b (all transcripts) |
| Abra | Alternaria brassicicola |
| Agam | Anopheles gambiae 49_3j (all transcripts) |
| Amel | Apis mellifera 37.2d (all transcripts) |
| Alyr | Arabidopsis lyrata |
| Atha | Arabidopsis thaliana 8 (all transcripts) |
| Agos | Ashbya gossypii ATCC 10895 |
| Acla | Aspergillus clavatus NRRL 1 |
| ASfla | Aspergillus flavus NRRL3357 |
| Afum2 | Aspergillus fumigatus A1163 |
| Afum | Aspergillus fumigatus Af293 |
| Anid | Aspergillus nidulans FGSC A4 |
| Anig | Aspergillus niger ATCC 1015 |
| Aory | Aspergillus oryzae RIB40 |
| Ater | Aspergillus terreus NIH2624 |
| Aano | Aureococcus anophagefferens |
| Bden2 | Batrachochytrium dendrobatidis JAM81 |
| Bden | Batrachochytrium dendrobatidis JEL423 |
| Bmor | Bombyx mori |
| Btau | Bos taurus 49_3f (all transcripts) |
| Bcin | Botrytis cinerea B05.10 |
| Bflo | Branchiostoma floridae 1.0 |
| BRmal | Brugia malayi |
| Cbre | Caenorhabditis brenneri |
| Cbri | Caenorhabditis briggsae 2 |
| Cele | Caenorhabditis elegans Ensembl 49_180a (all transcripts) |
| Cele2 | Caenorhabditis elegans WormBase WS194 (all transcripts) |
| CAjap | Caenorhabditis japonica |
| Crem | Caenorhabditis remanei |
| Calb | Candida albicans SC5314 |
| Calb2 | Candida albicans WO-1 |
| Cgla | Candida glabrata CBS138 |
| Clus | Candida lusitaniae ATCC 42720 |
| CApar | Candida parapsilosis |
| Ctro | Candida tropicalis MYA-3404 |
| Cfam | Canis familiaris 49_2g (all transcripts) |
| Capsp | Capitella sp. I |
| Cpap | Carica papaya |
| Cpor | Cavia porcellus 51_3 (all transcripts) |
| Cglo | Chaetomium globosum CBS 148.51 |
| Crei | Chlamydomonas reinhardtii 3.1 |
| Chlsp | Chlorella sp. NC64A |
| Cvul | Chlorella vulgaris |
| Cint | Ciona intestinalis 49_2i (all transcripts) |
| Csav | Ciona savignyi 49_2f (all transcripts) |
| Cimm2 | Coccidioides immitis H538.4 |
| Cimm3 | Coccidioides immitis RMSCC 2394 |
| Cimm4 | Coccidioides immitis RMSCC 3703 |
| Cimm | Coccidioides immitis RS |
| Cpos | Coccidioides posadasii RMSCC 3488 |
| Cpos2 | Coccidioides posadasii str. Silveira |
| Chet | Cochliobolus heterostrophus |
| Ccin | Coprinopsis cinerea okayama7 130 |
| CRHpar | Cryphonectria parasitica |
| Cneo2 | Cryptococcus neoformans B-3501A |
| Cneo | Cryptococcus neoformans JEC21 |
| Cneo3 | Cryptococcus neoformans var. grubii H99 |
| CRhom | Cryptosporidium hominis |
| CRmur | Cryptosporidium muris |
| CRTpar | Cryptosporidium parvum Iowa II |
| Cpip | Culex pipiens quinquefasciatus |
| Cmer | Cyanidioschyzon merolae |
| Drer | Danio rerio 49_7c (all transcripts) |
| Dpul | Daphnia pulex |
| Dnov | Dasypus novemcinctus 49_1f (all transcripts) |
| Dhan | Debaromyces hansenii |
| Ddis | Dictyostelium discoideum |
| Dpur | Dictyostelium purpureum |
| Dord | Dipodomys ordii 51_1 (all transcripts) |
| Dana | Drosophila ananassae 1.3 |
| Dere | Drosophila erecta 1.3 |
| Dgri | Drosophila grimshawi 1.3 |
| Dmel | Drosophila melanogaster Ensembl 49_54 (all transcripts) |
| Dmel2 | Drosophila melanogaster FlyBase 5.12 (all transcripts) |
| Dmoj | Drosophila mojavensis 1.3 |
| Dper | Drosophila persimilis 1.3 |
| Dpse | Drosophila pseudoobscura 2.3 |
| Dsec | Drosophila sechellia 1.3 |
| Dsim | Drosophila simulans 1.3 |
| Dvir | Drosophila virilis 1.2 |
| Dwil | Drosophila willistoni 1.3 |
| Dyak | Drosophila yakuba 1.3 |
| Etel | Echinops telfairi 49_1e (all transcripts) |
| Ehux | Emiliania huxleyi CCMP1516 |
| Ecun | Encephalitozoon cuniculi |
| Ehis | Entamoeba histolytica 1 |
| Ecab | Equus caballus 49_2 (all transcripts) |
| Eeur | Erinaceus europaeus 49_1c (all transcripts) |
| Fcat | Felis catus 49_1c (all transcripts) |
| Fgra | Fusarium graminearum |
| Foxy | Fusarium oxysporum f. sp. lycopersici 4286 |
| Fver | Fusarium verticillioides 7600 |
| Ggal | Gallus gallus 49_2g (all transcripts) |
| Gacu | Gasterosteus aculeatus 49_1f (all transcripts) |
| Glam | Giardia lamblia |
| Ggor | Gorilla gorilla 52_1 (all transcripts) |
| Hrob | Helobdella robusta |
| Hcap | Histoplasma capsulatum class NAmI strain WU24 |
| Hcap2 | Histoplasma capsulatum G186AR |
| Hcap3 | Histoplasma capsulatum H143 |
| Hcap4 | Histoplasma capsulatum H88 |
| Hsap | Homo sapiens 49_36k (all transcripts) |
| Klac | Kluyveromyces lactis |
| Kthe | Kluyveromyces thermotolerans CBS 6340 |
| Kwal | Kluyveromyces waltii |
| Lbic | Laccaria bicolor S238N-H82 |
| Lklu | Lachancea kluyveri |
| Lbra | Leishmania braziliensis MHOM/BR/75/M2904 |
| Linf | Leishmania infantum JPCM5 |
| Lmaj | Leishmania major strain Friedlin |
| Lelo | Lodderomyces elongisporus NRRL YB-4239 |
| Lgig | Lottia gigantea |
| Lafr | Loxodonta africana 49_1d (all transcripts) |
| Mmul | Macaca mulatta 49_10h (all transcripts) |
| Mgri | Magnaporthe grisea 70-15 |
| Mglo | Malassezia globosa CBS 7966 |
| Mtru | Medicago truncatula |
| Mlar | Melampsora laricis-populina |
| Mhap | Meloidogyne hapla |
| Mmur | Microcebus murinus 49_1 (all transcripts) |
| Micsp2 | Micromonas sp. CCMP490 |
| Micsp | Micromonas sp. RCC299 |
| Mgyp | Microsporum gypseum |
| Mdom | Monodelphis domestica 49_5d (all transcripts) |
| Mbre | Monosiga brevicollis |
| Mmus | Mus musculus 49_37b (all transcripts) |
| Mfij | Mycosphaerella fijiensis CIRAD86 |
| Mgra | Mycosphaerella graminicola IPO323 |
| Mluc | Myotis lucifugus 49_1e (all transcripts) |
| Ngru | Naegleria gruberi |
| Nvit | Nasonia vitripennis |
| Nhae | Nectria haematococca mpVI |
| Nvec | Nematostella vectensis 1.0 |
| Nfis | Neosartorya fischeri NRRL 181 |
| Ncan | Neospora caninum |
| Ncra | Neurospora crassa OR74A |
| Ndis | Neurospora discreta FGSC 8579 |
| Ntet | Neurospora tetrasperma |
| Opri | Ochotona princeps 49_1 (all transcripts) |
| Oana | Ornithorhynchus anatinus 49_1f (all transcripts) |
| Ocun | Oryctolagus cuniculus 49_1f (all transcripts) |
| Osat2 | Oryza sativa ssp. Indica |
| Osat | Oryza sativa ssp. japonica 5.0 |
| Olat | Oryzias latipes 49_1e (all transcripts) |
| Oluc | Ostreococcus lucimarinus CCE9901 |
| Ostsp | Ostreococcus sp. RCC809 |
| Otau | Ostreococcus tauri |
| Ogar | Otolemur garnettii 49_1c (all transcripts) |
| Ptro | Pan troglodytes 49_21h (all transcripts) |
| Pbra | Paracoccidioides brasiliensis Pb01 |
| Pbra2 | Paracoccidioides brasiliensis Pb03 |
| Pbra3 | Paracoccidioides brasiliensis Pb18 |
| Ptet | Paramecium tetraurelia |
| PHtri | Phaeodactylum tricornutum |
| Pchr | Phanerochaete chrysosporium RP-78 2.1 |
| Pbla | Phycomyces blakesleeanus |
| Ppat | Physcomitrella patens subsp. patens |
| PHcap | Phytophthora capsici |
| Pinf | Phytophthora infestans T30-4 |
| Pram | Phytophthora ramorum 1.1 |
| Psoj | Phytophthora sojae 1.1 |
| Psti | Pichia stipitis CBS 6054 |
| Pber | Plasmodium berghei ANKA |
| Pcha | Plasmodium chabaudi |
| Pfal | Plasmodium falciparum 3D7 |
| Pkno | Plasmodium knowlesi strain H |
| Pviv | Plasmodium vivax SaI-1 |
| Pyoe | Plasmodium yoelii ssp. yoelii 1 |
| Pans | Podospora anserina |
| Ppyg | Pongo pygmaeus 49_1 (all transcripts) |
| POtri | Populus trichocarpa 1.1 |
| Ppla | Postia placenta |
| Ppac | Pristionchus pacificus |
| PRcap | Procavia capensis 51_1 (all transcripts) |
| Pvam | Pteropus vampyrus 51_1 (all transcripts) |
| Pgra | Puccinia graminis f. sp. tritici CRL 75-36-700-3 |
| PYtri | Pyrenophora tritici-repentis |
| Rnor | Rattus norvegicus 49_34s (all transcripts) |
| Rory | Rhizopus oryzae RA 99-880 |
| Sbay | Saccharomyces bayanus MIT |
| Scer | Saccharomyces cerevisiae Ensembl 49_1h (all transcripts) |
| Scer3 | Saccharomyces cerevisiae RM11-1a |
| Scer2 | Saccharomyces cerevisiae SGD |
| Scer4 | Saccharomyces cerevisiae YJM789 |
| Smik | Saccharomyces mikatae MIT |
| Spar | Saccharomyces paradoxus MIT |
| Sjap | Schizosaccharomyces japonicus yFS275 |
| Soct | Schizosaccharomyces octosporus yFS286 |
| SCpom | Schizosaccharomyces pombe |
| SCpom2 | Schizosaccharomyces pombe 972h- |
| Sscl | Sclerotinia sclerotiorum |
| Smoe | Selaginella moellendorffii |
| Sara | Sorex araneus 49_1c (all transcripts) |
| Sbic | Sorghum bicolor |
| Stri | Spermophilus tridecemlineatus 49_1e (all transcripts) |
| Sros | Sporobolomyces roseus IAM 13481 |
| Snod | Stagonospora nodorum |
| Spur | Strongylocentrotus purpuratus |
| Trub | Takifugu rubripes 49_4i (all transcripts) |
| Tsyr | Tarsius syrichta 51_1 (all transcripts) |
| TEthe | Tetrahymena thermophila SB210 1 |
| Tnig | Tetraodon nigroviridis 49_1k (all transcripts) |
| THApse | Thalassiosira pseudonana |
| Tann | Theileria annulata |
| Tpar | Theileria parva |
| Tgon | Toxoplasma gondii ME49 |
| Tcas | Tribolium castaneum 3.0 |
| Tatr | Trichoderma atroviride |
| Tree | Trichoderma reesei 1.2 |
| Tvir | Trichoderma virens Gv29-8 |
| Tvag | Trichomonas vaginalis |
| Tadh | Trichoplax adhaerens |
| Tbru | Trypanosoma brucei |
| TRcru | Trypanosoma cruzi strain CL Brener |
| Tbel | Tupaia belangeri 49_1d (all transcripts) |
| Ttru | Tursiops truncatus 51_1 (all transcripts) |
| Uree | Uncinocarpus reesii 1704 |
| Umay | Ustilago maydis |
| Vpol | Vanderwaltozyma polyspora DSM 70294 |
| Valb | Verticillium albo-atrum VaMs.102 |
| Vdah | Verticillium dahliae VdLs.17 |
| Vpac | Vicugna pacos 51_1 (all transcripts) |
| Vvin | Vitis vinifera |
| Vcar | Volvox carteri f. nagariensis |
| Xlae | Xenopus laevis |
| Xtro | Xenopus tropicalis 49_41i (all transcripts) |
| Ylip | Yarrowia lipolytica CLIB122 |
| Zrou | Zygosaccharomyces rouxii |

Table S1-2 Archaea species list

| Abbreviation | Organism |
| --- | --- |
| Aper | Aeropyrum pernix K1 |
| Aful | Archaeoglobus fulgidus DSM 4304 |
| Cmaq | Caldivirga maquilingensis IC-167 |
| CKcry | Candidatus Korarchaeum cryptofilum OPF8 |
| CMboo | Candidatus Methanoregula boonei 6A8 |
| CMpal | Candidatus Methanosphaerula palustris E1-9c |
| Dkam | Desulfurococcus kamchatkensis 1221n |
| Hmar | Haloarcula marismortui ATCC 43049 |
| Hsal | Halobacterium salinarum R1 |
| Halsp | Halobacterium sp. NRC-1 |
| Hwal | Haloquadratum walsbyi DSM 16790 |
| Hbut | Hyperthermus butylicus DSM 5456 |
| Ihos | Ignicoccus hospitalis KIN4/I |
| Msed | Metallosphaera sedula DSM 5348 |
| Msmi | Methanobrevibacter smithii ATCC 35061 |
| Mjan | Methanocaldococcus jannaschii DSM 2661 |
| Mbur | Methanococcoides burtonii DSM 6242 |
| Maeo | Methanococcus aeolicus Nankai-3 |
| MEOmar2 | Methanococcus maripaludis C5 |
| MEOmar4 | Methanococcus maripaludis C6 |
| MEOmar3 | Methanococcus maripaludis C7 |
| MEOmar | Methanococcus maripaludis S2 |
| MEvan | Methanococcus vannielii SB |
| Mlab | Methanocorpusculum labreanum Z |
| MEUmar | Methanoculleus marisnigri JR1 |
| Mkan | Methanopyrus kandleri AV19 |
| MESthe | Methanosaeta thermophila PT |
| Mace | Methanosarcina acetivorans C2A |
| Mbar | Methanosarcina barkeri Fusaro |
| Mmaz | Methanosarcina mazei Go1 |
| Msta | Methanosphaera stadtmanae DSM 3091 |
| Mhun | Methanospirillum hungatei JF-1 |
| METthe | Methanothermobacter thermautotrophicus Delta H |
| Nequ | Nanoarchaeum equitans Kin4-M |
| Npha | Natronomonas pharaonis DSM 2160 |
| Nmar | Nitrosopumilus maritimus SCM1 |
| Ptor | Picrophilus torridus DSM 9790 |
| PYaer | Pyrobaculum aerophilum IM2 |
| Pars | Pyrobaculum arsenaticum DSM 13514 |
| Pcal | Pyrobaculum calidifontis JCM 11548 |
| Pisl | Pyrobaculum islandicum DSM 4184 |
| Paby | Pyrococcus abyssi GE5 |
| Pfur | Pyrococcus furiosus DSM 3638 |
| Phor | Pyrococcus horikoshii OT3 |
| Smar | Staphylothermus marinus F1 |
| SUaci | Sulfolobus acidocaldarius DSM 639 |
| Ssol | Sulfolobus solfataricus P2 |
| Stok | Sulfolobus tokodaii 7 |
| Tkod | Thermococcus kodakarensis KOD1 |
| Tonn | Thermococcus onnurineus NA1 |
| Tpen | Thermofilum pendens Hrk 5 |
| Taci | Thermoplasma acidophilum DSM 1728 |
| Tvol | Thermoplasma volcanium GSS1 |
| Tneu | Thermoproteus neutrophilus V24Sta |

Table S1-3 Bacteria species list

| Abbreviation | Organism |
| --- | --- |
| ACmar | Acaryochloris marina MBIC11017 |
| Alai | Acholeplasma laidlawii PG-8A |
| Abac | Acidobacteria bacterium Ellin345 |
| Acel | Acidothermus cellulolyticus 11B |
| Aave | Acidovorax avenae ssp. citrulli AAC00-1 |
| Acidsp | Acidovorax sp. JS42 |
| Abau2 | Acinetobacter baumannii AB0057 |
| Abau3 | Acinetobacter baumannii AB307-0294 |
| Abau4 | Acinetobacter baumannii ACICU |
| Abau | Acinetobacter baumannii ATCC 17978 |
| Abau5 | Acinetobacter baumannii AYE |
| Abau6 | Acinetobacter baumannii SDF |
| Acinsp | Acinetobacter sp. ADP1 |
| Aple | Actinobacillus pleuropneumoniae L20 |
| Aple2 | Actinobacillus pleuropneumoniae ser. 3 JL03 |
| Aple3 | Actinobacillus pleuropneumoniae ser. 7 AP76 |
| Asuc | Actinobacillus succinogenes 130Z |
| Ahyd | Aeromonas hydrophila ssp. hydrophila ATCC 7966 |
| AEsal | Aeromonas salmonicida ssp. salmonicida A449 |
| Atum | Agrobacterium tumefaciens C58 |
| Amuc | Akkermansia muciniphila ATCC BAA-835 |
| Abor | Alcanivorax borkumensis SK2 |
| ALsal | Aliivibrio salmonicida LFI1238 |
| Aehr | Alkalilimnicola ehrlichei MLHE-1 |
| Amet | Alkaliphilus metalliredigens QYMF |
| Aore | Alkaliphilus oremlandii OhILAs |
| Amac | Alteromonas macleodii Deep ecotype |
| Avar | Anabaena variabilis ATCC 29413 |
| Adeh | Anaeromyxobacter dehalogenans 2CP-C |
| Anasp | Anaeromyxobacter sp. Fw109-5 |
| Anasp2 | Anaeromyxobacter sp. K |
| ANmar | Anaplasma marginale St. Maries |
| Apha | Anaplasma phagocytophilum HZ |
| ANfla | Anoxybacillus flavithermus WK1 |
| Aaeo | Aquifex aeolicus VF5 |
| Abut | Arcobacter butzleri RM4018 |
| Aaro | Aromatoleum aromaticum EbN1 |
| Aaur | Arthrobacter aurescens TC1 |
| Artsp | Arthrobacter sp. FB24 |
| Ayel | Aster yellows witches-broom phytoplasma AYWB |
| Azosp | Azoarcus sp. BH72 |
| Acau | Azorhizobium caulinodans ORS 571 |
| Bamy | Bacillus amyloliquefaciens FZB42 |
| Bant2 | Bacillus anthracis Ames |
| Bant | Bacillus anthracis Ames Ancestor |
| Bant3 | Bacillus anthracis Sterne |
| Bcer5 | Bacillus cereus AH187 |
| Bcer6 | Bacillus cereus AH820 |
| Bcer2 | Bacillus cereus ATCC 10987 |
| Bcer | Bacillus cereus ATCC 14579 |
| Bcer3 | Bacillus cereus E33L |
| Bcer7 | Bacillus cereus G9842 |
| Bcer4 | Bacillus cereus ssp. cytotoxis NVH 391-98 |
| Bcla | Bacillus clausii KSM-K16 |
| Bhal | Bacillus halodurans C-125 |
| Blic | Bacillus licheniformis ATCC 14580 |
| Bpum | Bacillus pumilus SAFR-032 |
| Bsub | Bacillus subtilis ssp. subtilis 168 |
| Bthu2 | Bacillus thuringiensis Al Hakam |
| Bthu | Bacillus thuringiensis ser. konkukian 97-27 |
| Bwei | Bacillus weihenstephanensis KBAB4 |
| Bfra | Bacteroides fragilis NCTC 9343 |
| Bfra2 | Bacteroides fragilis YCH46 |
| Bthe | Bacteroides thetaiotaomicron VPI-5482 |
| Bvul | Bacteroides vulgatus ATCC 8482 |
| BAbac | Bartonella bacilliformis KC583 |
| Bhen | Bartonella henselae Houston-1 |
| Bqui | Bartonella quintana Toulouse |
| Btri | Bartonella tribocorum CIP 105476 |
| Bcic | Baumannia cicadellinicola Hc |
| BDbac | Bdellovibrio bacteriovorus HD100 |
| Bind | Beijerinckia indica ssp. indica ATCC 9039 |
| Bado | Bifidobacterium adolescentis ATCC 15703 |
| Bani | Bifidobacterium animalis ssp. lactis AD011 |
| Blon2 | Bifidobacterium longum |
| Blon | Bifidobacterium longum NCC2705 |
| Blon3 | Bifidobacterium longum ssp. infantis ATCC 15697 |
| Bavi | Bordetella avium 197N |
| Bbro | Bordetella bronchiseptica RB50 |
| Bpar | Bordetella parapertussis 12822 |
| Bper | Bordetella pertussis Tohama I |
| Bpet | Bordetella petrii DSM 12804 |
| Bafz | Borrelia afzelii PKo |
| Bbur | Borrelia burgdorferi B31 |
| Bbur2 | Borrelia burgdorferi ZS7 |
| Bdut | Borrelia duttonii Ly |
| Bgar | Borrelia garinii PBi |
| Bher | Borrelia hermsii DAH |
| Brec | Borrelia recurrentis A1 |
| Btur | Borrelia turicatae 91E135 |
| Bjap | Bradyrhizobium japonicum USDA 110 |
| Brasp2 | Bradyrhizobium sp. BTAi1 |
| Brasp | Bradyrhizobium sp. ORS278 |
| Babo | Brucella abortus bv. 1 9-941 |
| Babo2 | Brucella abortus S19 |
| Bcan | Brucella canis ATCC 23365 |
| Bmel | Brucella melitensis 16M |
| Bmel2 | Brucella melitensis biov. Abortus 2308 |
| Bovi | Brucella ovis ATCC 25840 |
| Bsui | Brucella suis 1330 |
| Bsui2 | Brucella suis ATCC 23445 |
| Baph5 | Buchnera aphidicola 5A |
| Baph | Buchnera aphidicola APS |
| Baph2 | Buchnera aphidicola Bp |
| Baph4 | Buchnera aphidicola Cc |
| Baph3 | Buchnera aphidicola Sg |
| Baph6 | Buchnera aphidicola Tuc7 |
| Bamb | Burkholderia ambifaria AMMD |
| Bamb2 | Burkholderia ambifaria MC40-6 |
| Bcen | Burkholderia cenocepacia AU 1054 |
| Bcen2 | Burkholderia cenocepacia HI2424 |
| Bcen3 | Burkholderia cenocepacia J2315 |
| Bcen4 | Burkholderia cenocepacia MC0-3 |
| BUmal | Burkholderia mallei ATCC 23344 |
| BUmal2 | Burkholderia mallei NCTC 10229 |
| BUmal3 | Burkholderia mallei NCTC 10247 |
| BUmal4 | Burkholderia mallei SAVP1 |
| Bmul | Burkholderia multivorans ATCC 17616 |
| Bphym | Burkholderia phymatum STM815 |
| Bphyt | Burkholderia phytofirmans PsJN |
| Bpse2 | Burkholderia pseudomallei 1106a |
| Bpse3 | Burkholderia pseudomallei 1710b |
| Bpse4 | Burkholderia pseudomallei 668 |
| Bpse | Burkholderia pseudomallei K96243 |
| Bursp | Burkholderia sp. 383 |
| Btha | Burkholderia thailandensis E264 |
| Bvie | Burkholderia vietnamiensis G4 |
| Bxen | Burkholderia xenovorans LB400 |
| Csac | Caldicellulosiruptor saccharolyticus DSM 8903 |
| Ccon | Campylobacter concisus 13826 |
| Ccur | Campylobacter curvus 525.92 |
| Cfet | Campylobacter fetus ssp. fetus 82-40 |
| CAhom | Campylobacter hominis ATCC BAA-381 |
| Cjej2 | Campylobacter jejuni RM1221 |
| Cjej3 | Campylobacter jejuni ssp. doylei 269.97 |
| Cjej5 | Campylobacter jejuni ssp. jejuni 81116 |
| Cjej4 | Campylobacter jejuni ssp. jejuni 81-176 |
| Cjej | Campylobacter jejuni ssp. jejuni NCTC 11168 |
| CAasi | Candidatus Amoebophilus asiaticus 5a2 |
| CApse | Candidatus Azobacteroides pseudotrichonymphae genomovar. CFP2 |
| CBflo | Candidatus Blochmannia floridanus |
| CBpen | Candidatus Blochmannia pennsylvanicus BPEN |
| CCrud | Candidatus Carsonella ruddii PV |
| CDaud | Candidatus Desulforudis audaxviator MP104C |
| CPubi | Candidatus Pelagibacter ubique HTCC1062 |
| CPaus | Candidatus Phytoplasma australiense |
| CPmal | Candidatus Phytoplasma mali |
| CPamo | Candidatus Protochlamydia amoebophila UWE25 |
| CRmag | Candidatus Ruthia magnifica Cm |
| CSmue | Candidatus Sulcia muelleri GWSS |
| CVoku | Candidatus Vesicomyosocius okutanii HA |
| Chyd | Carboxydothermus hydrogenoformans Z-2901 |
| Ccre | Caulobacter crescentus CB15 |
| Causp | Caulobacter sp. K31 |
| CEjap | Cellvibrio japonicus Ueda107 |
| CHmur | Chlamydia muridarum Nigg |
| Ctra3 | Chlamydia trachomatis 434/Bu |
| Ctra2 | Chlamydia trachomatis A/HAR-13 |
| Ctra | Chlamydia trachomatis D/UW-3/CX |
| Ctra4 | Chlamydia trachomatis L2b/UCH-1/proctitis |
| Cabo | Chlamydophila abortus S26/3 |
| Ccav | Chlamydophila caviae GPIC |
| Cfel | Chlamydophila felis Fe/C-56 |
| Cpne4 | Chlamydophila pneumoniae AR39 |
| Cpne2 | Chlamydophila pneumoniae CWL029 |
| Cpne3 | Chlamydophila pneumoniae J138 |
| Cpne | Chlamydophila pneumoniae TW-183 |
| CHpar | Chlorobaculum parvum NCIB 8327 |
| Cchl | Chlorobium chlorochromatii CaD3 |
| Clim | Chlorobium limicola DSM 245 |
| Cphab2 | Chlorobium phaeobacteroides BS1 |
| Cphab | Chlorobium phaeobacteroides DSM 266 |
| Cphav | Chlorobium phaeovibrioides DSM 265 |
| Ctep | Chlorobium tepidum TLS |
| Cagg | Chloroflexus aggregans DSM 9485 |
| Caur | Chloroflexus aurantiacus J-10-fl |
| Ctha | Chloroherpeton thalassium ATCC 35110 |
| Cvio | Chromobacterium violaceum ATCC 12472 |
| Csal | Chromohalobacter salexigens DSM 3043 |
| Ckos | Citrobacter koseri ATCC BAA-895 |
| Cmic | Clavibacter michiganensis ssp. michiganensis NCPPB 382 |
| Cmic2 | Clavibacter michiganensis ssp. sepedonicus |
| Cace | Clostridium acetobutylicum ATCC 824 |
| Cbei | Clostridium beijerinckii NCIMB 8052 |
| Cbot2 | Clostridium botulinum A ATCC 19397 |
| Cbot | Clostridium botulinum A ATCC 3502 |
| Cbot3 | Clostridium botulinum A Hall |
| Cbot5 | Clostridium botulinum A3 Loch Maree |
| Cbot6 | Clostridium botulinum B Eklund 17B |
| Cbot7 | Clostridium botulinum B1 Okra |
| Cbot8 | Clostridium botulinum E3 Alaska E43 |
| Cbot4 | Clostridium botulinum F Langeland |
| Cdif | Clostridium difficile 630 |
| Cklu | Clostridium kluyveri DSM 555 |
| Cnov | Clostridium novyi NT |
| Cper | Clostridium perfringens 13 |
| Cper2 | Clostridium perfringens ATCC 13124 |
| Cper3 | Clostridium perfringens SM101 |
| Cphy | Clostridium phytofermentans ISDg |
| Ctet | Clostridium tetani E88 |
| Cthe | Clostridium thermocellum ATCC 27405 |
| Cpsy | Colwellia psychrerythraea 34H |
| Cpro | Coprothermobacter proteolyticus DSM 5265 |
| Cdip | Corynebacterium diphtheriae NCTC 13129 |
| Ceff | Corynebacterium efficiens YS-314 |
| Cglu | Corynebacterium glutamicum ATCC 13032 Kitasato |
| Cglu3 | Corynebacterium glutamicum R |
| Cjei | Corynebacterium jeikeium K411 |
| Cure | Corynebacterium urealyticum DSM 7109 |
| Cbur4 | Coxiella burnetii CbuG_Q212 |
| Cbur5 | Coxiella burnetii CbuK_Q154 |
| Cbur2 | Coxiella burnetii Dugway 5J108-111 |
| Cbur3 | Coxiella burnetii RSA 331 |
| Cbur | Coxiella burnetii RSA 493 |
| Ctai | Cupriavidus taiwanensis |
| Cyasp | Cyanothece sp. ATCC 51142 |
| Cyasp2 | Cyanothece sp. PCC 7424 |
| Cyasp3 | Cyanothece sp. PCC 8801 |
| Chut | Cytophaga hutchinsonii ATCC 33406 |
| Daro | Dechloromonas aromatica RCB |
| Deth | Dehalococcoides ethenogenes 195 |
| Dehsp2 | Dehalococcoides sp. BAV1 |
| Dehsp | Dehalococcoides sp. CBDB1 |
| Dgeo | Deinococcus geothermalis DSM 11300 |
| Drad | Deinococcus radiodurans R1 |
| Daci | Delftia acidovorans SPH-1 |
| Dalk | Desulfatibacillum alkenivorans AK-01 |
| Dhaf2 | Desulfitobacterium hafniense DCB-2 |
| Dhaf | Desulfitobacterium hafniense Y51 |
| Dole | Desulfococcus oleovorans Hxd3 |
| Dpsy | Desulfotalea psychrophila LSv54 |
| Dred | Desulfotomaculum reducens MI-1 |
| Ddes | Desulfovibrio desulfuricans ssp. desulfuricans G20 |
| Dvul2 | Desulfovibrio vulgaris DP4 |
| Dvul | Desulfovibrio vulgaris Hildenborough |
| Dvul3 | Desulfovibrio vulgaris Miyazaki F |
| Dnod | Dichelobacter nodosus VCS1703A |
| Dthe | Dictyoglomus thermophilum H-6-12 |
| Dtur | Dictyoglomus turgidum DSM 6724 |
| Dshi | Dinoroseobacter shibae DFL 12 |
| Ecan | Ehrlichia canis Jake |
| Echa | Ehrlichia chaffeensis Arkansas |
| Erum2 | Ehrlichia ruminantium Gardel |
| Erum | Ehrlichia ruminantium Welgevonden |
| Emin | Elusimicrobium minutum Pei191 |
| Esak | Enterobacter sakazakii ATCC BAA-894 |
| Entsp | Enterobacter sp. 638 |
| Efae | Enterococcus faecalis V583 |
| Etas | Erwinia tasmaniensis Et1/99 |
| Elit | Erythrobacter litoralis HTCC2594 |
| Ecol5 | Escherichia coli 536 |
| Ecol11 | Escherichia coli 55989 |
| Ecol6 | Escherichia coli APEC O1 |
| Ecol12 | Escherichia coli ATCC 8739 |
| Ecol2 | Escherichia coli CFT073 |
| Ecol9 | Escherichia coli E24377A |
| Ecol13 | Escherichia coli ED1a |
| Ecol10 | Escherichia coli HS |
| Ecol14 | Escherichia coli IAI1 |
| Ecol15 | Escherichia coli IAI39 |
| Ecol16 | Escherichia coli K12 |
| Ecol17 | Escherichia coli K12 subDH10B |
| Ecol8 | Escherichia coli K-12 subW3110 |
| Ecol18 | Escherichia coli O127:H6 E2348/69 |
| Ecol19 | Escherichia coli O157:H7 EC4115 |
| Ecol4 | Escherichia coli O157:H7 EDL933 |
| Ecol3 | Escherichia coli O157:H7 Sakai |
| Ecol20 | Escherichia coli S88 |
| Ecol21 | Escherichia coli SE11 |
| Ecol22 | Escherichia coli SMS-3-5 |
| Ecol23 | Escherichia coli SMS-3-5 |
| Ecol24 | Escherichia coli UMN026 |
| Ecol7 | Escherichia coli UTI89 |
| Efer | Escherichia fergusonii ATCC 35469 |
| Esib | Exiguobacterium sibiricum 255-15 |
| Fnod | Fervidobacterium nodosum Rt17-B1 |
| Fmag | Finegoldia magna ATCC 29328 |
| Fjoh | Flavobacterium johnsoniae UW101 |
| Fpsy | Flavobacterium psychrophilum JIP02/86 |
| Fnov | Francisella novicida U112 |
| Fphi | Francisella philomiragia ssp. philomiragia ATCC 25017 |
| Ftul2 | Francisella tularensis ssp. holarctica |
| Ftul7 | Francisella tularensis ssp. holarctica FTNF002-00 |
| Ftul3 | Francisella tularensis ssp. holarctica OSU18 |
| Ftul8 | Francisella tularensis ssp. mediasiatica FSC147 |
| Ftul5 | Francisella tularensis ssp. tularensis FSC198 |
| Ftul | Francisella tularensis ssp. tularensis SCHU S4 |
| Ftul6 | Francisella tularensis ssp. tularensis WY96-3418 |
| Faln | Frankia alni ACN14a |
| Frasp | Frankia sp. CcI3 |
| Frasp2 | Frankia sp. EAN1pec |
| Fnuc | Fusobacterium nucleatum ssp. nucleatum ATCC 25586 |
| Gkau | Geobacillus kaustophilus HTA426 |
| Gthe | Geobacillus thermodenitrificans NG80-2 |
| Gbem | Geobacter bemidjiensis Bem |
| Glov | Geobacter lovleyi SZ |
| Gmet | Geobacter metallireducens GS-15 |
| Gsul | Geobacter sulfurreducens PCA |
| Gura | Geobacter uraniireducens Rf4 |
| Gvio | Gloeobacter violaceus PCC 7421 |
| Gdia | Gluconacetobacter diazotrophicus PAl 5 |
| Goxy | Gluconobacter oxydans 621H |
| Gfor | Gramella forsetii KT0803 |
| Gbet | Granulibacter bethesdensis CGDNIH1 |
| Hduc | Haemophilus ducreyi 35000HP |
| Hinf2 | Haemophilus influenzae 86-028NP |
| Hinf3 | Haemophilus influenzae PittEE |
| Hinf4 | Haemophilus influenzae PittGG |
| Hinf | Haemophilus influenzae Rd KW20 |
| Hsom | Haemophilus somnus 129PT |
| Hsom2 | Haemophilus somnus 2336 |
| Hche | Hahella chejuensis KCTC 2396 |
| Hhal | Halorhodospira halophila SL1 |
| Haci | Helicobacter acinonychis Sheeba |
| Hhep | Helicobacter hepaticus ATCC 51449 |
| Hpyl | Helicobacter pylori 26695 |
| Hpyl4 | Helicobacter pylori G27 |
| Hpyl3 | Helicobacter pylori HPAG1 |
| Hpyl2 | Helicobacter pylori J99 |
| Hpyl5 | Helicobacter pylori P12 |
| Hpyl6 | Helicobacter pylori Shi470 |
| Hmod | Heliobacterium modesticaldum Ice1 |
| Hars | Herminiimonas arsenicoxydans |
| Haur | Herpetosiphon aurantiacus ATCC 23779 |
| Hydsp | Hydrogenobaculum sp. Y04AAS1 |
| Hnep | Hyphomonas neptunium ATCC 15444 |
| Iloi | Idiomarina loihiensis L2TR |
| Jannsp | Jannaschia sp. CCS1 |
| Jantsp | Janthinobacterium sp. Marseille |
| Krad | Kineococcus radiotolerans SRS30216 |
| Kpne2 | Klebsiella pneumoniae 342 |
| Kpne | Klebsiella pneumoniae ssp. pneumoniae MGH 78578 |
| Krhi | Kocuria rhizophila DC2201 |
| Laci | Lactobacillus acidophilus NCFM |
| Lbre | Lactobacillus brevis ATCC 367 |
| Lcas | Lactobacillus casei ATCC 334 |
| Lcas2 | Lactobacillus casei BL23 |
| Ldel | Lactobacillus delbrueckii ssp. bulgaricus ATCC 11842 |
| Ldel2 | Lactobacillus delbrueckii ssp. bulgaricus ATCC BAA-365 |
| Lfer | Lactobacillus fermentum IFO 3956 |
| Lgas | Lactobacillus gasseri ATCC 33323 |
| Lhel | Lactobacillus helveticus DPC 4571 |
| Ljoh | Lactobacillus johnsonii NCC 533 |
| Lpla | Lactobacillus plantarum WCFS1 |
| Lreu | Lactobacillus reuteri DSM 20016 |
| Lreu2 | Lactobacillus reuteri JCM 1112 |
| Lsak | Lactobacillus sakei ssp. sakei 23K |
| Lsal | Lactobacillus salivarius UCC118 |
| Llac2 | Lactococcus lactis ssp. cremoris MG1363 |
| Llac3 | Lactococcus lactis ssp. cremoris SK11 |
| Llac | Lactococcus lactis ssp. lactis Il1403 |
| LAint | Lawsonia intracellularis PHE/MN1-00 |
| Lpne4 | Legionella pneumophila Corby |
| Lpne2 | Legionella pneumophila Lens |
| Lpne3 | Legionella pneumophila Paris |
| Lpne | Legionella pneumophila ssp. pneumophila Philadelphia 1 |
| Lxyl | Leifsonia xyli ssp. xyli CTCB07 |
| Lbif2 | Leptospira biflexa ser. Patoc Patoc 1 (Ames) |
| Lbif | Leptospira biflexa ser. Patoc Patoc 1 (Paris) |
| Lbor2 | Leptospira borgpetersenii ser. Hardjo-bovis JB197 |
| Lbor | Leptospira borgpetersenii ser. Hardjo-bovis L550 |
| LEint2 | Leptospira interrogans ser. Copenhageni Fiocruz L1-130 |
| LEint | Leptospira interrogans ser. Lai 56601 |
| Lcho | Leptothrix cholodnii SP-6 |
| Lcit | Leuconostoc citreum KM20 |
| Lmes | Leuconostoc mesenteroides ssp. mesenteroides ATCC 8293 |
| Linn | Listeria innocua Clip11262 |
| Lmon2 | Listeria monocytogenes 4b F2365 |
| Lmon | Listeria monocytogenes EGD-e |
| Lmon3 | Listeria monocytogenes HCC23 |
| Lwel | Listeria welshimeri ser. 6b SLCC5334 |
| Lsph | Lysinibacillus sphaericus C3-41 |
| Magsp | Magnetococcus sp. MC-1 |
| Mmag | Magnetospirillum magneticum AMB-1 |
| Msuc | Mannheimia succiniciproducens MBEL55E |
| MAmar | Maricaulis maris MCS10 |
| Maqu | Marinobacter aquaeolei VT8 |
| Marsp | Marinomonas sp. MWYL1 |
| Mflo | Mesoplasma florum L1 |
| Mlot | Mesorhizobium loti MAFF303099 |
| Messp | Mesorhizobium sp. BNC1 |
| Minf | Methylacidiphilum infernorum V4 |
| Mpet | Methylibium petroleiphilum PM1 |
| Mfla | Methylobacillus flagellatus KT |
| Mchl | Methylobacterium chloromethanicum CM4 |
| Mext | Methylobacterium extorquens PA1 |
| Mpop | Methylobacterium populi BJ001 |
| Mrad | Methylobacterium radiotolerans JCM 2831 |
| Metsp | Methylobacterium sp. 4-46 |
| Msil | Methylocella silvestris BL2 |
| MEcap | Methylococcus capsulatus Bath |
| Maer | Microcystis aeruginosa NIES-843 |
| MOthe | Moorella thermoacetica ATCC 39073 |
| Mabs | Mycobacterium abscessus |
| Mavi2 | Mycobacterium avium 104 |
| Mavi | Mycobacterium avium ssp. paratuberculosis K-10 |
| Mbov | Mycobacterium bovis AF2122/97 |
| Mbov2 | Mycobacterium bovis BCG Pasteur 1173P2 |
| Mgil | Mycobacterium gilvum PYR-GCK |
| Mlep | Mycobacterium leprae TN |
| MYmar | Mycobacterium marinum M |
| Msme | Mycobacterium smegmatis MC2 155 |
| Mycsp2 | Mycobacterium sp. JLS |
| Mycsp3 | Mycobacterium sp. KMS |
| Mycsp | Mycobacterium sp. MCS |
| Mtub2 | Mycobacterium tuberculosis CDC1551 |
| Mtub3 | Mycobacterium tuberculosis F11 |
| Mtub4 | Mycobacterium tuberculosis H37Ra |
| Mtub | Mycobacterium tuberculosis H37Rv |
| Mulc | Mycobacterium ulcerans Agy99 |
| MYvan | Mycobacterium vanbaalenii PYR-1 |
| Maga | Mycoplasma agalactiae PG2 |
| Mart | Mycoplasma arthritidis 158L3-1 |
| MYcap | Mycoplasma capricolum ssp. capricolum ATCC 27343 |
| Mgal | Mycoplasma gallisepticum R |
| Mgen | Mycoplasma genitalium G37 |
| Mhyo | Mycoplasma hyopneumoniae 232 |
| Mhyo2 | Mycoplasma hyopneumoniae 7448 |
| Mhyo3 | Mycoplasma hyopneumoniae J |
| Mmob | Mycoplasma mobile 163K |
| Mmyc | Mycoplasma mycoides ssp. mycoides SC PG1 |
| Mpen | Mycoplasma penetrans HF-2 |
| Mpne | Mycoplasma pneumoniae M129 |
| Mpul | Mycoplasma pulmonis UAB CTIP |
| Msyn | Mycoplasma synoviae 53 |
| Mxan | Myxococcus xanthus DK 1622 |
| Nthe | Natranaerobius thermophilus JW/NM-WN-LF |
| Ngon | Neisseria gonorrhoeae FA 1090 |
| Ngon2 | Neisseria gonorrhoeae NCCP11945 |
| Nmen4 | Neisseria meningitidis 053442 |
| Nmen3 | Neisseria meningitidis FAM18 |
| Nmen2 | Neisseria meningitidis MC58 |
| Nmen | Neisseria meningitidis Z2491 |
| Nsen | Neorickettsia sennetsu Miyayama |
| Nitsp | Nitratiruptor sp. SB155-2 |
| Nham | Nitrobacter hamburgensis X14 |
| Nwin | Nitrobacter winogradskyi Nb-255 |
| Noce | Nitrosococcus oceani ATCC 19707 |
| Neur | Nitrosomonas europaea ATCC 19718 |
| Neut | Nitrosomonas eutropha C91 |
| Nmul | Nitrosospira multiformis ATCC 25196 |
| Nfar | Nocardia farcinica IFM 10152 |
| Nocsp | Nocardioides sp. JS614 |
| Npun | Nostoc punctiforme PCC 73102 |
| Nossp | Nostoc sp. PCC 7120 |
| Naro | Novosphingobium aromaticivorans DSM 12444 |
| Oihe | Oceanobacillus iheyensis HTE831 |
| Oant | Ochrobactrum anthropi ATCC 49188 |
| Ooen | Oenococcus oeni PSU-1 |
| Ocar | Oligotropha carboxidovorans OM5 |
| Oyel | Onion yellows phytoplasma OY-M |
| Oter | Opitutus terrae PB90-1 |
| Otsu | Orientia tsutsugamushi Boryong |
| Otsu2 | Orientia tsutsugamushi Ikeda |
| Pdis | Parabacteroides distasonis ATCC 8503 |
| Pden | Paracoccus denitrificans PD1222 |
| Plav | Parvibaculum lavamentivorans DS-1 |
| Pmul | Pasteurella multocida ssp. multocida Pm70 |
| Patr | Pectobacterium atrosepticum SCRI1043 |
| Ppen | Pediococcus pentosaceus ATCC 25745 |
| Pcar | Pelobacter carbinolicus DSM 2380 |
| PEpro | Pelobacter propionicus DSM 2379 |
| Plut | Pelodictyon luteolum DSM 273 |
| Ppha | Pelodictyon phaeoclathratiforme BU-1 |
| Pthe | Pelotomaculum thermopropionicum SI |
| Pmob | Petrotoga mobilis SJ95 |
| PHpro | Photobacterium profundum SS9 |
| Plum | Photorhabdus luminescens ssp. laumondii TTO1 |
| Pnap | Polaromonas naphthalenivorans CJ2 |
| Polsp | Polaromonas sp. JS666 |
| Pnec | Polynucleobacter necessarius ssp. asymbioticus QLW-P1DMWA-1 |
| Pnec2 | Polynucleobacter necessarius ssp. necessarius STIR1 |
| Pgin2 | Porphyromonas gingivalis ATCC 33277 |
| Pgin | Porphyromonas gingivalis W83 |
| Pmar5 | Prochlorococcus marinus AS9601 |
| Pmar11 | Prochlorococcus marinus MIT 9211 |
| Pmar12 | Prochlorococcus marinus MIT 9215 |
| Pmar6 | Prochlorococcus marinus MIT 9301 |
| Pmar7 | Prochlorococcus marinus MIT 9303 |
| Pmar8 | Prochlorococcus marinus MIT 9312 |
| Pmar | Prochlorococcus marinus MIT 9313 |
| Pmar9 | Prochlorococcus marinus MIT 9515 |
| Pmar10 | Prochlorococcus marinus NATL1A |
| Pmar4 | Prochlorococcus marinus NATL2A |
| Pmar2 | Prochlorococcus marinus ssp. marinus CCMP1375 |
| Pmar3 | Prochlorococcus marinus ssp. pastoris CCMP1986 |
| Pacn | Propionibacterium acnes KPA171202 |
| Paes | Prosthecochloris aestuarii DSM 271 |
| Pmir | Proteus mirabilis HI4320 |
| Patl | Pseudoalteromonas atlantica T6c |
| Phal | Pseudoalteromonas haloplanktis TAC125 |
| PSaer4 | Pseudomonas aeruginosa LESB58 |
| PSaer2 | Pseudomonas aeruginosa PA7 |
| PSaer | Pseudomonas aeruginosa PAO1 |
| PSaer3 | Pseudomonas aeruginosa UCBPP-PA14 |
| Pent | Pseudomonas entomophila L48 |
| Pflu2 | Pseudomonas fluorescens Pf0-1 |
| Pflu | Pseudomonas fluorescens Pf-5 |
| Pmen | Pseudomonas mendocina ymp |
| Pput2 | Pseudomonas putida F1 |
| Pput3 | Pseudomonas putida GB-1 |
| Pput | Pseudomonas putida KT2440 |
| Pstu | Pseudomonas stutzeri A1501 |
| Psyr2 | Pseudomonas syringae pv. phaseolicola 1448A |
| Psyr3 | Pseudomonas syringae pv. syringae B728a |
| Psyr | Pseudomonas syringae pv. tomato DC3000 |
| Parc | Psychrobacter arcticus 273-4 |
| Pcry | Psychrobacter cryohalolentis K5 |
| Psysp | Psychrobacter sp. PRwf-1 |
| Ping | Psychromonas ingrahamii 37 |
| Reut2 | Ralstonia eutropha H16 |
| Reut | Ralstonia eutropha JMP134 |
| Rmet | Ralstonia metallidurans CH34 |
| Rsol | Ralstonia solanacearum GMI1000 |
| Rsal | Renibacterium salmoninarum ATCC 33209 |
| Retl | Rhizobium etli CFN 42 |
| Retl2 | Rhizobium etli CIAT 652 |
| Rleg2 | Rhizobium leguminosarum bv. trifolii WSM2304 |
| Rleg | Rhizobium leguminosarum bv. viciae 3841 |
| Rsph | Rhodobacter sphaeroides 2.4.1 |
| Rsph2 | Rhodobacter sphaeroides ATCC 17025 |
| Rsph3 | Rhodobacter sphaeroides ATCC 17029 |
| Rjos | Rhodococcus jostii RHA1 |
| Rfer | Rhodoferax ferrireducens T118 |
| Rbal | Rhodopirellula baltica SH 1 |
| Rpal2 | Rhodopseudomonas palustris BisA53 |
| Rpal3 | Rhodopseudomonas palustris BisB18 |
| Rpal4 | Rhodopseudomonas palustris BisB5 |
| Rpal | Rhodopseudomonas palustris CGA009 |
| Rpal5 | Rhodopseudomonas palustris HaA2 |
| Rpal6 | Rhodopseudomonas palustris TIE-1 |
| Rrub | Rhodospirillum rubrum ATCC 11170 |
| Raka | Rickettsia akari Hartford |
| Rbel2 | Rickettsia bellii OSU 85-389 |
| Rbel | Rickettsia bellii RML369-C |
| Rcan | Rickettsia canadensis McKiel |
| Rcon | Rickettsia conorii Malish 7 |
| Rfel | Rickettsia felis URRWXCal2 |
| Rmas | Rickettsia massiliae MTU5 |
| Rpro | Rickettsia prowazekii Madrid E |
| Rric2 | Rickettsia rickettsii Iowa |
| Rric | Rickettsia rickettsii Sheila Smith |
| Rtyp | Rickettsia typhi Wilmington |
| Rcas | Roseiflexus castenholzii DSM 13941 |
| Rossp | Roseiflexus sp. RS-1 |
| Rden | Roseobacter denitrificans OCh 114 |
| Rxyl | Rubrobacter xylanophilus DSM 9941 |
| Sdeg | Saccharophagus degradans 2-40 |
| Sery | Saccharopolyspora erythraea NRRL 2338 |
| Srub | Salinibacter ruber DSM 13855 |
| Sare | Salinispora arenicola CNS-205 |
| Stro | Salinispora tropica CNB-440 |
| Sent5 | Salmonella enterica ssp. arizonae ser. 62:z4,z23:-- |
| Sent7 | Salmonella enterica ssp. enterica ser. Agona SL483 |
| Sent3 | Salmonella enterica ssp. enterica ser. Choleraesuis str. SC-B67 |
| Sent8 | Salmonella enterica ssp. enterica ser. Dublin CT_02021853 |
| Sent9 | Salmonella enterica ssp. enterica ser. Enteritidis str. |
| Sent10 | Salmonella enterica ssp. enterica ser. Gallinarum 287/91 |
| Sent11 | Salmonella enterica ssp. enterica ser. Heidelberg SL476 |
| Sent12 | Salmonella enterica ssp. enterica ser. Newport SL254 |
| Sent | Salmonella enterica ssp. enterica ser. Paratyphi A ATCC 9150 |
| Sent6 | Salmonella enterica ssp. enterica ser. Paratyphi B SPB7 |
| Sent13 | Salmonella enterica ssp. enterica ser. Schwarzengrund str. |
| Sent4 | Salmonella enterica ssp. enterica ser. Typhi CT18 |
| Sent2 | Salmonella enterica ssp. enterica ser. Typhi Ty2 |
| Stypm | Salmonella typhimurium LT2 |
| Spro | Serratia proteamaculans 568 |
| Sama | Shewanella amazonensis SB2B |
| Sbal | Shewanella baltica OS155 |
| Sbal2 | Shewanella baltica OS185 |
| Sbal3 | Shewanella baltica OS195 |
| Sbal4 | Shewanella baltica OS223 |
| SHden | Shewanella denitrificans OS217 |
| Sfri | Shewanella frigidimarina NCIMB 400 |
| Shal | Shewanella halifaxensis HAW-EB4 |
| Sloi | Shewanella loihica PV-4 |
| Sone | Shewanella oneidensis MR-1 |
| Spea | Shewanella pealeana ATCC 700345 |
| Spie | Shewanella piezotolerans WP3 |
| Sput | Shewanella putrefaciens CN-32 |
| Ssed | Shewanella sediminis HAW-EB3 |
| Shesp2 | Shewanella sp. ANA-3 |
| Shesp3 | Shewanella sp. MR-4 |
| Shesp | Shewanella sp. MR-7 |
| Shesp4 | Shewanella sp. W3-18-1 |
| Swoo | Shewanella woodyi ATCC 51908 |
| Sboy2 | Shigella boydii CDC 3083-94 |
| Sboy | Shigella boydii Sb227 |
| Sdys | Shigella dysenteriae Sd197 |
| Sfle | Shigella flexneri 2a 2457T |
| Sfle2 | Shigella flexneri 2a 301 |
| Sfle3 | Shigella flexneri 5 8401 |
| Sson | Shigella sonnei Ss046 |
| SIpom | Silicibacter pomeroyi DSS-3 |
| Silsp | Silicibacter sp. TM1040 |
| Smed | Sinorhizobium medicae WSM419 |
| Smel | Sinorhizobium meliloti 1021 |
| Sglo | Sodalis glossinidius morsitans |
| Susi | Solibacter usitatus Ellin6076 |
| Scel | Sorangium cellulosum So ce 56 |
| Swit | Sphingomonas wittichii RW1 |
| Sala | Sphingopyxis alaskensis RB2256 |
| Saur14 | Staphylococcus aureus RF122 |
| Saur6 | Staphylococcus aureus ssp. aureus COL |
| Saur7 | Staphylococcus aureus ssp. aureus JH1 |
| Saur8 | Staphylococcus aureus ssp. aureus JH9 |
| Saur9 | Staphylococcus aureus ssp. aureus MRSA252 |
| Saur2 | Staphylococcus aureus ssp. aureus MSSA476 |
| Saur13 | Staphylococcus aureus ssp. aureus Mu3 |
| Saur3 | Staphylococcus aureus ssp. aureus Mu50 |
| Saur4 | Staphylococcus aureus ssp. aureus MW2 |
| Saur5 | Staphylococcus aureus ssp. aureus N315 |
| Saur | Staphylococcus aureus ssp. aureus NCTC 8325 |
| Saur10 | Staphylococcus aureus ssp. aureus Newman |
| Saur11 | Staphylococcus aureus ssp. aureus USA300 |
| Saur12 | Staphylococcus aureus ssp. aureus USA300_TCH1516 |
| Sepi2 | Staphylococcus epidermidis ATCC 12228 |
| Sepi | Staphylococcus epidermidis RP62A |
| Shae | Staphylococcus haemolyticus JCSC1435 |
| Ssap | Staphylococcus saprophyticus ssp. saprophyticus ATCC 15305 |
| Smal2 | Stenotrophomonas maltophilia K279a |
| Smal | Stenotrophomonas maltophilia R551-3 |
| Saga2 | Streptococcus agalactiae 2603V/R |
| Saga3 | Streptococcus agalactiae A909 |
| Saga | Streptococcus agalactiae NEM316 |
| Sequ | Streptococcus equi ssp. zooepidemicus MGCS10565 |
| Sgor | Streptococcus gordonii Challis subCH1 |
| Smut | Streptococcus mutans UA159 |
| Spne4 | Streptococcus pneumoniae CGSP14 |
| Spne3 | Streptococcus pneumoniae D39 |
| Spne5 | Streptococcus pneumoniae G54 |
| Spne6 | Streptococcus pneumoniae Hungary19A-6 |
| Spne2 | Streptococcus pneumoniae R6 |
| Spne | Streptococcus pneumoniae TIGR4 |
| Spyo | Streptococcus pyogenes M1 GAS |
| Spyo8 | Streptococcus pyogenes Manfredo |
| Spyo9 | Streptococcus pyogenes MGAS10270 |
| Spyo5 | Streptococcus pyogenes MGAS10394 |
| Spyo10 | Streptococcus pyogenes MGAS10750 |
| Spyo11 | Streptococcus pyogenes MGAS2096 |
| Spyo2 | Streptococcus pyogenes MGAS315 |
| Spyo6 | Streptococcus pyogenes MGAS5005 |
| Spyo7 | Streptococcus pyogenes MGAS6180 |
| Spyo3 | Streptococcus pyogenes MGAS8232 |
| Spyo12 | Streptococcus pyogenes MGAS9429 |
| Spyo13 | Streptococcus pyogenes NZ131 |
| Spyo4 | Streptococcus pyogenes SSI-1 |
| Ssan | Streptococcus sanguinis SK36 |
| Ssui | Streptococcus suis 05ZYH33 |
| Ssui2 | Streptococcus suis 98HAH33 |
| STthe2 | Streptococcus thermophilus CNRZ1066 |
| STthe3 | Streptococcus thermophilus LMD-9 |
| STthe | Streptococcus thermophilus LMG 18311 |
| Save | Streptomyces avermitilis MA-4680 |
| Scoe | Streptomyces coelicolor A3(2) |
| Sgri | Streptomyces griseus ssp. griseus NBRC 13350 |
| Sulisp | Sulfurihydrogenibium sp. YO3AOP1 |
| SDden | Sulfurimonas denitrificans DSM 1251 |
| Sulosp | Sulfurovum sp. NBC37-1 |
| SYthe | Symbiobacterium thermophilum IAM 14863 |
| Selo | Synechococcus elongatus PCC 6301 |
| Selo2 | Synechococcus elongatus PCC 7942 |
| Synosp2 | Synechococcus sp. CC9311 |
| Synosp5 | Synechococcus sp. PCC 7002 |
| Synosp3 | Synechococcus sp. RCC307 |
| Synosp4 | Synechococcus sp. WH 7803 |
| Synosp | Synechococcus sp. WH 8102 |
| Synysp | Synechocystis sp. PCC 6803 |
| Sfum | Syntrophobacter fumaroxidans MPOB |
| Swol | Syntrophomonas wolfei ssp. wolfei Goettingen |
| SYaci | Syntrophus aciditrophicus SB |
| THEpse | Thermoanaerobacter pseudethanolicus ATCC 33223 |
| Theasp | Thermoanaerobacter sp. X514 |
| Tten | Thermoanaerobacter tengcongensis MB4 |
| Tfus | Thermobifida fusca YX |
| Tyel | Thermodesulfovibrio yellowstonii DSM 11347 |
| Tafr | Thermosipho africanus TCF52B |
| Tmel | Thermosipho melanesiensis BI429 |
| Telo | Thermosynechococcus elongatus BP-1 |
| Tlet | Thermotoga lettingae TMO |
| Tmar | Thermotoga maritima MSB8 |
| Tpet | Thermotoga petrophila RKU-1 |
| Thetsp | Thermotoga sp. RQ2 |
| THthe | Thermus thermophilus HB27 |
| THthe2 | Thermus thermophilus HB8 |
| THden | Thiobacillus denitrificans ATCC 25259 |
| THcru | Thiomicrospira crunogena XCL-2 |
| TRden | Treponema denticola ATCC 35405 |
| Tpal | Treponema pallidum ssp. pallidum Nichols |
| Tpal2 | Treponema pallidum ssp. pallidum SS14 |
| Tery | Trichodesmium erythraeum IMS101 |
| Twhi2 | Tropheryma whipplei TW08/27 |
| Twhi | Tropheryma whipplei Twist |
| Upar2 | Ureaplasma parvum ser. 3 ATCC 27815 |
| Upar | Ureaplasma parvum ser. 3 ATCC 700970 |
| Veis | Verminephrobacter eiseniae EF01-2 |
| Vcho | Vibrio cholerae O1 biov. eltor N16961 |
| Vcho2 | Vibrio cholerae O395 |
| Vfis | Vibrio fischeri ES114 |
| Vfis2 | Vibrio fischeri MJ11 |
| Vhar | Vibrio harveyi ATCC BAA-1116 |
| Vpar | Vibrio parahaemolyticus RIMD 2210633 |
| Vvul | Vibrio vulnificus YJ016 |
| Wglo | Wigglesworthia glossinidia |
| Wpip | Wolbachia pipientis |
| Wsuc | Wolinella succinogenes DSM 1740 |
| Xaut | Xanthobacter autotrophicus Py2 |
| Xaxo | Xanthomonas axonopodis pv. citri 306 |
| Xcam2 | Xanthomonas campestris pv. campestris 8004 |
| Xcam | Xanthomonas campestris pv. campestris ATCC 33913 |
| Xcam4 | Xanthomonas campestris pv. campestris B100 |
| Xcam3 | Xanthomonas campestris pv. vesicatoria 85-10 |
| Xory | Xanthomonas oryzae pv. oryzae KACC10331 |
| Xory2 | Xanthomonas oryzae pv. oryzae MAFF 311018 |
| Xory3 | Xanthomonas oryzae pv. oryzae PXO99A |
| Xfas | Xylella fastidiosa 9a5c |
| Xfas3 | Xylella fastidiosa M12 |
| Xfas4 | Xylella fastidiosa M23 |
| Xfas2 | Xylella fastidiosa Temecula1 |
| Yent | Yersinia enterocolitica ssp. enterocolitica 8081 |
| Ypes7 | Yersinia pestis Angola |
| Ypes4 | Yersinia pestis Antiqua |
| Ypes2 | Yersinia pestis biov. Microtus 91001 |
| Ypes | Yersinia pestis CO92 |
| Ypes3 | Yersinia pestis KIM |
| Ypes5 | Yersinia pestis Nepal516 |
| Ypes6 | Yersinia pestis Pestoides F |
| Ypse2 | Yersinia pseudotuberculosis IP 31758 |
| Ypse | Yersinia pseudotuberculosis IP 32953 |
| Ypse3 | Yersinia pseudotuberculosis PB1/+ |
| Ypse4 | Yersinia pseudotuberculosis YPIII |
| Zmob | Zymomonas mobilis ssp. mobilis ZM4 |
