# Supplementary material for: The Evolutionary History of Protein Domains Viewed by Species Phylogeny
Source: PLoS One. 2009 Dec 21;4(12):e8378. doi: 10.1371/journal.pone.0008378 (PMC2794708; doi:10.1371/journal.pone.0008378)
Supplement: Table S2 — Predicted number of protein domains originated from each ancestor node in the tree of life with respect to different Rhgt values. (0.49 MB DOC) [file pone.0008378.s005.doc]

Table S2. Predicted number of protein domains originated from each ancestor node in the tree of life with respect to different R_hgt_ values.

|  |  | Rhgt |  |  |  |  |  |  |  |  |  |  |  |  |
| --- | --- | --- | --- | --- | --- | --- | --- | --- | --- | --- | --- | --- | --- | --- |
|  | level within the tree | 3 | 4 | 5 | 6 | 7 | 8 | 9 | 10 | 11 | 12 | 13 | 14 | 15 |
| Total |  | 26393 | 18657 | 14002 | 11068 | 9200 | 7850 | 7121 | 6327 | 5747 | 5402 | 5078 | 4709 | 4403 |
| cellular organisms | 22 | 1303 | 1527 | 1643 | 1740 | 1828 | 1890 | 1931 | 1984 | 2027 | 2056 | 2083 | 2111 | 2140 |
| Eukaryota | 21 | 620 | 582 | 559 | 551 | 529 | 513 | 504 | 492 | 489 | 485 | 485 | 482 | 472 |
| Fungi/Metazoa group | 20 | 100 | 76 | 67 | 58 | 52 | 46 | 45 | 37 | 31 | 33 | 33 | 33 | 32 |
| Metazoa | 19 | 90 | 78 | 72 | 72 | 67 | 64 | 59 | 58 | 50 | 47 | 45 | 43 | 41 |
| Eumetazoa | 18 | 88 | 78 | 75 | 61 | 54 | 50 | 48 | 47 | 47 | 42 | 39 | 36 | 34 |
| Bilateria | 17 | 24 | 31 | 28 | 23 | 23 | 22 | 21 | 17 | 19 | 19 | 17 | 17 | 15 |
| Coelomata | 16 | 32 | 20 | 24 | 25 | 22 | 22 | 22 | 20 | 19 | 19 | 19 | 17 | 17 |
| Deuterostomia | 15 | 8 | 6 | 5 | 5 | 5 | 5 | 5 | 5 | 5 | 5 | 5 | 5 | 5 |
| Chordata | 14 | 21 | 19 | 16 | 15 | 15 | 15 | 15 | 15 | 15 | 13 | 12 | 11 | 11 |
| Euteleostomi | 13 | 36 | 33 | 32 | 27 | 24 | 20 | 19 | 19 | 19 | 19 | 19 | 19 | 19 |
| Tetrapoda | 12 | 5 | 5 | 5 | 1 | 2 | 2 | 2 | 2 | 2 | 1 | 1 | 1 | 1 |
| Amniota | 11 | 7 | 5 | 5 | 5 | 4 | 4 | 4 | 3 | 3 | 3 | 3 | 3 | 3 |
| Bacteria | 10 | 248 | 195 | 186 | 181 | 164 | 154 | 151 | 144 | 129 | 119 | 108 | 97 | 93 |
| Mammalia | 10 | 4 | 3 | 3 | 3 | 4 | 3 | 2 | 2 | 2 | 2 | 2 | 2 | 2 |
| Fungi | 10 | 26 | 23 | 18 | 13 | 13 | 11 | 11 | 10 | 8 | 7 | 5 | 4 | 4 |
| Protostomia | 10 | 6 | 5 | 3 | 2 | 2 | 3 | 2 | 1 | 1 | 1 | 1 | 1 | 1 |
| Dikarya | 9 | 36 | 23 | 29 | 25 | 24 | 21 | 18 | 14 | 13 | 12 | 11 | 9 | 9 |
| Pancrustacea | 9 | 12 | 11 | 6 | 6 | 6 | 6 | 6 | 6 | 5 | 5 | 5 | 5 | 5 |
| Viridiplantae | 9 | 82 | 54 | 44 | 29 | 24 | 23 | 12 | 9 | 5 | 5 | 4 | 4 | 4 |
| Proteobacteria | 9 | 170 | 157 | 135 | 109 | 101 | 99 | 96 | 87 | 78 | 77 | 79 | 82 | 79 |
| Theria | 9 | 2 | 2 | 2 | 1 | 1 | 1 | 1 | 0 | 0 | 0 | 0 | 0 | 0 |
| Neoptera | 8 | 1 | 2 | 3 | 3 | 1 | 1 | 1 | 1 | 1 | 1 | 1 | 1 | 1 |
| Embryophyta | 8 | 60 | 54 | 48 | 34 | 29 | 27 | 24 | 15 | 14 | 9 | 8 | 8 | 7 |
| delta/epsilon subdivisions | 8 | 13 | 4 | 3 | 2 | 3 | 3 | 2 | 2 | 2 | 3 | 4 | 4 | 4 |
| Alphaproteobacteria | 8 | 42 | 26 | 22 | 17 | 16 | 11 | 9 | 8 | 9 | 9 | 9 | 8 | 7 |
| Actinobacteria (class) | 8 | 26 | 11 | 9 | 7 | 4 | 3 | 2 | 1 | 0 | 0 | 0 | 0 | 0 |
| Ascomycota | 8 | 12 | 12 | 7 | 5 | 4 | 5 | 3 | 3 | 3 | 3 | 3 | 5 | 4 |
| Firmicutes | 8 | 53 | 35 | 33 | 25 | 28 | 35 | 32 | 28 | 27 | 26 | 25 | 26 | 25 |
| Eutheria | 8 | 9 | 7 | 9 | 9 | 5 | 3 | 4 | 5 | 4 | 4 | 1 | 1 | 1 |
| Tracheophyta | 7 | 24 | 20 | 18 | 13 | 12 | 9 | 8 | 8 | 5 | 5 | 4 | 3 | 3 |
| Betaproteobacteria | 7 | 14 | 10 | 10 | 7 | 8 | 3 | 3 | 2 | 2 | 2 | 2 | 2 | 2 |
| Clostridia | 7 | 28 | 16 | 13 | 7 | 5 | 3 | 2 | 3 | 1 | 0 | 0 | 0 | 0 |
| Euarchontoglires | 7 | 6 | 2 | 3 | 3 | 0 | 0 | 0 | 0 | 0 | 0 | 0 | 0 | 0 |
| Rickettsiales | 7 | 1 | 1 | 1 | 2 | 2 | 1 | 1 | 1 | 1 | 0 | 0 | 0 | 0 |
| Archaea | 7 | 36 | 19 | 15 | 17 | 16 | 15 | 14 | 13 | 12 | 8 | 8 | 9 | 9 |
| Actinobacteridae | 7 | 18 | 16 | 13 | 8 | 3 | 3 | 1 | 1 | 1 | 1 | 0 | 0 | 0 |
| Bacilli | 7 | 38 | 31 | 27 | 17 | 16 | 10 | 9 | 7 | 9 | 9 | 10 | 10 | 12 |
| Pezizomycotina | 7 | 99 | 92 | 43 | 37 | 21 | 18 | 16 | 15 | 14 | 11 | 11 | 9 | 9 |
| Endopterygota | 7 | 9 | 6 | 5 | 5 | 3 | 2 | 1 | 1 | 1 | 1 | 1 | 1 | 1 |
| Epsilonproteobacteria | 7 | 7 | 5 | 2 | 1 | 1 | 1 | 1 | 1 | 1 | 1 | 1 | 1 | 1 |
| Gammaproteobacteria | 7 | 49 | 45 | 38 | 36 | 28 | 29 | 26 | 22 | 21 | 20 | 18 | 19 | 19 |
| Diptera | 6 | 4 | 2 | 2 | 3 | 2 | 2 | 2 | 2 | 1 | 1 | 1 | 1 | 1 |
| Burkholderiales | 6 | 77 | 29 | 17 | 12 | 6 | 3 | 2 | 2 | 1 | 2 | 1 | 1 | 1 |
| Magnoliophyta | 6 | 22 | 22 | 57 | 50 | 35 | 29 | 25 | 24 | 22 | 18 | 15 | 12 | 11 |
| Spirochaetales | 6 | 10 | 3 | 2 | 0 | 0 | 0 | 0 | 0 | 0 | 0 | 0 | 0 | 0 |
| Pseudomonadales | 6 | 25 | 16 | 9 | 4 | 2 | 3 | 2 | 1 | 1 | 1 | 1 | 2 | 1 |
| Rhizobiales | 6 | 40 | 33 | 15 | 13 | 7 | 3 | 5 | 6 | 7 | 7 | 4 | 2 | 2 |
| Clostridiales | 6 | 9 | 8 | 3 | 7 | 4 | 3 | 3 | 2 | 2 | 2 | 2 | 1 | 1 |
| Bacillales | 6 | 40 | 26 | 15 | 17 | 12 | 11 | 9 | 9 | 9 | 10 | 10 | 10 | 9 |
| Lactobacillales | 6 | 25 | 16 | 13 | 10 | 4 | 3 | 3 | 3 | 3 | 3 | 3 | 3 | 4 |
| Campylobacterales | 6 | 1 | 1 | 1 | 1 | 1 | 0 | 0 | 0 | 1 | 1 | 1 | 1 | 1 |
| Bacteroidetes/Chlorobi group | 6 | 19 | 12 | 7 | 5 | 5 | 0 | 0 | 0 | 0 | 0 | 0 | 0 | 0 |
| Alveolata | 6 | 4 | 1 | 0 | 0 | 0 | 0 | 0 | 0 | 0 | 0 | 0 | 0 | 0 |
| Primates | 6 | 3 | 3 | 2 | 2 | 0 | 0 | 0 | 0 | 0 | 0 | 0 | 0 | 0 |
| Eurotiomycetidae | 6 | 12 | 5 | 6 | 5 | 3 | 1 | 1 | 1 | 1 | 1 | 1 | 1 | 1 |
| Euryarchaeota | 6 | 76 | 32 | 29 | 17 | 17 | 18 | 17 | 14 | 13 | 13 | 12 | 12 | 12 |
| Chlamydiae/Verrucomicrobia group | 6 | 10 | 5 | 4 | 1 | 1 | 0 | 0 | 0 | 0 | 0 | 0 | 0 | 0 |
| Rickettsiaceae | 6 | 3 | 4 | 3 | 2 | 2 | 1 | 1 | 0 | 0 | 0 | 0 | 0 | 0 |
| Actinomycetales | 6 | 101 | 78 | 62 | 42 | 28 | 25 | 27 | 19 | 16 | 14 | 14 | 15 | 14 |
| core eudicotyledons | 5 | 5 | 5 | 3 | 3 | 2 | 2 | 2 | 2 | 2 | 2 | 1 | 1 | 1 |
| Chloroflexi | 5 | 7 | 5 | 3 | 2 | 1 | 1 | 1 | 1 | 1 | 1 | 1 | 1 | 1 |
| Campylobacteraceae | 5 | 0 | 0 | 0 | 0 | 0 | 0 | 0 | 0 | 0 | 0 | 0 | 0 | 0 |
| Spirochaetaceae | 5 | 3 | 1 | 2 | 0 | 0 | 0 | 0 | 0 | 0 | 0 | 0 | 0 | 0 |
| Thermoprotei | 5 | 16 | 7 | 6 | 5 | 6 | 6 | 5 | 4 | 3 | 3 | 2 | 2 | 2 |
| Corynebacterineae | 5 | 24 | 19 | 13 | 17 | 14 | 12 | 5 | 3 | 2 | 1 | 1 | 1 | 1 |
| Burkholderiaceae | 5 | 24 | 14 | 17 | 13 | 8 | 4 | 3 | 1 | 2 | 1 | 1 | 1 | 1 |
| Mollicutes | 5 | 9 | 7 | 4 | 3 | 3 | 2 | 2 | 2 | 2 | 2 | 2 | 2 | 2 |
| Chlorobiaceae | 5 | 35 | 21 | 10 | 7 | 6 | 5 | 4 | 2 | 2 | 2 | 2 | 1 | 1 |
| Sordariomycetes | 5 | 13 | 13 | 11 | 12 | 13 | 7 | 5 | 2 | 2 | 2 | 2 | 2 | 2 |
| Pseudomonadaceae | 5 | 13 | 3 | 2 | 2 | 1 | 0 | 0 | 0 | 0 | 0 | 0 | 0 | 0 |
| Cyanobacteria | 5 | 110 | 99 | 75 | 61 | 54 | 52 | 45 | 43 | 39 | 37 | 37 | 36 | 33 |
| Basidiomycota | 5 | 3 | 1 | 4 | 3 | 2 | 2 | 2 | 2 | 1 | 1 | 1 | 1 | 1 |
| Thiotrichales | 5 | 2 | 1 | 1 | 1 | 1 | 0 | 0 | 0 | 0 | 0 | 0 | 0 | 0 |
| Chlamydiales | 5 | 2 | 2 | 2 | 2 | 2 | 2 | 1 | 1 | 0 | 0 | 0 | 0 | 0 |
| Glires | 5 | 0 | 3 | 2 | 2 | 2 | 2 | 0 | 0 | 0 | 0 | 0 | 0 | 0 |
| Streptococcaceae | 5 | 1 | 1 | 2 | 2 | 2 | 2 | 2 | 2 | 2 | 2 | 2 | 1 | 1 |
| Haplorrhini | 5 | 1 | 1 | 1 | 1 | 1 | 0 | 0 | 0 | 0 | 0 | 0 | 0 | 0 |
| Apicomplexa | 5 | 3 | 3 | 1 | 0 | 0 | 0 | 0 | 0 | 0 | 0 | 0 | 0 | 0 |
| Rhizobiaceae | 5 | 14 | 4 | 4 | 1 | 0 | 0 | 0 | 0 | 0 | 0 | 0 | 0 | 0 |
| Onygenales | 5 | 13 | 10 | 8 | 6 | 4 | 4 | 4 | 4 | 2 | 1 | 1 | 1 | 1 |
| Saccharomycetales | 5 | 4 | 3 | 3 | 3 | 2 | 2 | 2 | 2 | 2 | 2 | 2 | 2 | 2 |
| Enterobacteriaceae | 5 | 93 | 65 | 62 | 50 | 44 | 41 | 37 | 32 | 31 | 26 | 25 | 21 | 24 |
| Methanomicrobia | 5 | 35 | 27 | 22 | 9 | 13 | 12 | 12 | 2 | 1 | 0 | 0 | 0 | 0 |
| Deltaproteobacteria | 5 | 48 | 23 | 16 | 14 | 15 | 12 | 10 | 11 | 10 | 9 | 8 | 6 | 5 |
| Bacteroidetes | 5 | 19 | 16 | 13 | 13 | 12 | 8 | 5 | 2 | 2 | 0 | 0 | 0 | 0 |
| Clostridiaceae | 5 | 19 | 10 | 2 | 0 | 0 | 0 | 1 | 2 | 1 | 1 | 1 | 1 | 1 |
| Bacillaceae | 5 | 51 | 17 | 9 | 9 | 7 | 7 | 7 | 6 | 6 | 6 | 6 | 5 | 5 |
| Rickettsieae | 5 | 4 | 4 | 3 | 2 | 1 | 1 | 1 | 1 | 0 | 0 | 0 | 0 | 0 |
| Salmonella enterica | 4 | 31 | 18 | 16 | 16 | 11 | 10 | 9 | 9 | 9 | 9 | 9 | 9 | 6 |
| Chlamydiaceae | 4 | 4 | 4 | 3 | 3 | 3 | 3 | 3 | 2 | 2 | 2 | 2 | 2 | 2 |
| mitosporic Onygenales | 4 | 0 | 0 | 0 | 0 | 0 | 0 | 0 | 0 | 0 | 0 | 0 | 0 | 0 |
| rosids | 4 | 6 | 6 | 6 | 6 | 6 | 4 | 4 | 2 | 2 | 2 | 2 | 2 | 2 |
| Trichocomaceae | 4 | 15 | 10 | 5 | 9 | 5 | 3 | 2 | 2 | 2 | 2 | 2 | 2 | 2 |
| Agaricomycotina | 4 | 5 | 2 | 1 | 0 | 0 | 0 | 0 | 0 | 0 | 0 | 0 | 0 | 0 |
| Neisseriaceae | 4 | 8 | 2 | 0 | 0 | 0 | 0 | 0 | 0 | 0 | 0 | 0 | 0 | 0 |
| Staphylococcus | 4 | 18 | 13 | 5 | 5 | 4 | 3 | 2 | 2 | 2 | 2 | 2 | 2 | 2 |
| Acholeplasmataceae | 4 | 0 | 0 | 0 | 0 | 0 | 0 | 0 | 0 | 0 | 0 | 0 | 0 | 0 |
| Micrococcineae | 4 | 5 | 7 | 5 | 3 | 2 | 1 | 0 | 0 | 0 | 0 | 0 | 0 | 0 |
| Helicobacteraceae | 4 | 1 | 1 | 1 | 1 | 1 | 1 | 1 | 1 | 1 | 1 | 1 | 1 | 1 |
| Pasteurellaceae | 4 | 24 | 16 | 13 | 10 | 9 | 6 | 5 | 5 | 5 | 4 | 4 | 4 | 4 |
| Rhodobacterales | 4 | 5 | 1 | 2 | 3 | 1 | 1 | 1 | 1 | 1 | 1 | 1 | 1 | 0 |
| Hypocreales | 4 | 31 | 18 | 13 | 10 | 8 | 6 | 5 | 2 | 2 | 0 | 0 | 0 | 0 |
| Alteromonadales | 4 | 44 | 26 | 21 | 14 | 7 | 5 | 6 | 4 | 3 | 2 | 2 | 2 | 2 |
| Sordariomycetidae | 4 | 5 | 5 | 2 | 0 | 0 | 0 | 0 | 0 | 0 | 0 | 0 | 0 | 0 |
| Chloroflexi (class) | 4 | 61 | 29 | 18 | 11 | 9 | 7 | 3 | 3 | 3 | 3 | 2 | 1 | 1 |
| Campylobacter | 4 | 3 | 2 | 2 | 2 | 0 | 0 | 0 | 0 | 0 | 0 | 0 | 0 | 0 |
| Catarrhini | 4 | 10 | 6 | 6 | 5 | 3 | 3 | 3 | 1 | 1 | 1 | 1 | 1 | 1 |
| Vibrionaceae | 4 | 46 | 28 | 18 | 13 | 6 | 5 | 4 | 2 | 2 | 1 | 1 | 1 | 1 |
| Myxococcales | 4 | 88 | 54 | 20 | 15 | 9 | 9 | 3 | 2 | 2 | 2 | 2 | 2 | 2 |
| Dothideomycetes | 4 | 2 | 2 | 2 | 1 | 0 | 0 | 0 | 0 | 0 | 0 | 0 | 0 | 0 |
| Chlorophyta | 4 | 21 | 14 | 13 | 7 | 4 | 4 | 4 | 1 | 1 | 1 | 0 | 0 | 0 |
| Clupeocephala | 4 | 0 | 0 | 0 | 0 | 0 | 0 | 0 | 0 | 0 | 0 | 0 | 0 | 0 |
| Aconoidasida | 4 | 2 | 1 | 1 | 1 | 1 | 1 | 1 | 1 | 1 | 1 | 0 | 0 | 0 |
| Methanosarcinales | 4 | 9 | 7 | 4 | 3 | 3 | 2 | 2 | 2 | 0 | 0 | 0 | 0 | 0 |
| Thermoanaerobacterales | 4 | 2 | 0 | 0 | 0 | 0 | 0 | 0 | 0 | 0 | 0 | 0 | 0 | 0 |
| Francisella | 4 | 21 | 14 | 11 | 6 | 4 | 3 | 3 | 3 | 3 | 3 | 2 | 2 | 1 |
| Mycoplasmataceae | 4 | 0 | 0 | 0 | 0 | 0 | 0 | 0 | 0 | 0 | 0 | 0 | 0 | 0 |
| Rhizobium/Agrobacterium group | 4 | 11 | 6 | 6 | 4 | 3 | 3 | 3 | 2 | 2 | 1 | 1 | 0 | 0 |
| Bacillus | 4 | 40 | 34 | 56 | 39 | 24 | 13 | 13 | 10 | 9 | 9 | 8 | 8 | 7 |
| Escherichia | 4 | 32 | 27 | 18 | 19 | 10 | 5 | 5 | 4 | 4 | 4 | 4 | 3 | 0 |
| Shigella | 4 | 42 | 35 | 20 | 17 | 10 | 2 | 2 | 2 | 2 | 2 | 2 | 1 | 0 |
| Mycobacterium | 4 | 35 | 27 | 22 | 17 | 15 | 15 | 14 | 18 | 11 | 11 | 11 | 9 | 8 |
| Desulfovibrionaceae | 4 | 4 | 4 | 2 | 1 | 0 | 0 | 0 | 0 | 0 | 0 | 0 | 0 | 0 |
| Streptococcus | 4 | 27 | 15 | 7 | 6 | 8 | 8 | 8 | 8 | 6 | 5 | 4 | 4 | 3 |
| Bacteroidales | 4 | 27 | 15 | 8 | 7 | 4 | 4 | 4 | 4 | 3 | 2 | 2 | 2 | 2 |
| Saccharomycetaceae | 4 | 9 | 4 | 4 | 4 | 3 | 3 | 2 | 2 | 2 | 2 | 2 | 2 | 2 |
| Flavobacteriales | 4 | 0 | 0 | 0 | 0 | 0 | 0 | 0 | 0 | 0 | 0 | 0 | 0 | 0 |
| Trypanosomatidae | 4 | 21 | 15 | 10 | 7 | 4 | 4 | 3 | 3 | 3 | 3 | 3 | 3 | 3 |
| Burkholderia | 4 | 83 | 65 | 37 | 20 | 19 | 18 | 15 | 15 | 5 | 5 | 4 | 3 | 3 |
| Brucellaceae | 4 | 7 | 4 | 4 | 4 | 4 | 3 | 3 | 2 | 2 | 1 | 1 | 1 | 1 |
| Anaplasmataceae | 4 | 1 | 1 | 1 | 0 | 0 | 0 | 0 | 0 | 0 | 0 | 0 | 0 | 0 |
| Rickettsia | 4 | 10 | 7 | 7 | 7 | 6 | 2 | 2 | 2 | 2 | 2 | 2 | 2 | 2 |
| Lactobacillaceae | 4 | 2 | 1 | 0 | 0 | 0 | 0 | 0 | 0 | 0 | 0 | 0 | 0 | 0 |
| Pseudomonas | 4 | 61 | 55 | 46 | 37 | 29 | 22 | 18 | 15 | 13 | 13 | 13 | 5 | 3 |
| Moraxellaceae | 4 | 5 | 1 | 1 | 0 | 0 | 0 | 0 | 0 | 0 | 0 | 0 | 0 | 0 |
| Xanthomonadaceae | 4 | 45 | 31 | 17 | 13 | 8 | 5 | 3 | 1 | 1 | 0 | 0 | 0 | 0 |
| Methanococcales | 4 | 10 | 5 | 4 | 2 | 1 | 1 | 1 | 0 | 0 | 0 | 0 | 0 | 0 |
| Sophophora | 4 | 8 | 6 | 5 | 6 | 3 | 3 | 2 | 2 | 2 | 1 | 1 | 1 | 1 |
| Chlorobium/Pelodictyon group | 4 | 4 | 3 | 9 | 5 | 5 | 4 | 4 | 3 | 2 | 2 | 1 | 1 | 0 |
| Thermoproteales | 4 | 13 | 7 | 2 | 2 | 0 | 0 | 0 | 0 | 0 | 0 | 0 | 0 | 0 |
| Clostridium | 4 | 42 | 58 | 43 | 23 | 9 | 3 | 3 | 3 | 4 | 5 | 5 | 4 | 1 |
| Borrelia | 4 | 9 | 3 | 2 | 2 | 2 | 2 | 2 | 2 | 1 | 1 | 1 | 1 | 1 |
| Chroococcales | 4 | 27 | 10 | 8 | 4 | 0 | 0 | 0 | 0 | 0 | 0 | 0 | 0 | 0 |
| Rodentia | 4 | 3 | 1 | 1 | 1 | 1 | 1 | 1 | 1 | 1 | 1 | 1 | 1 | 1 |
| Desulfovibrio | 3 | 6 | 5 | 4 | 0 | 0 | 0 | 0 | 0 | 0 | 0 | 0 | 0 | 0 |
| Filobasidiella neoformans | 3 | 5 | 4 | 1 | 1 | 1 | 1 | 1 | 0 | 0 | 0 | 0 | 0 | 0 |
| Lactobacillus | 3 | 11 | 5 | 3 | 2 | 2 | 1 | 1 | 1 | 1 | 1 | 1 | 1 | 1 |
| Mycobacterium tuberculosis complex | 3 | 35 | 28 | 19 | 16 | 15 | 13 | 13 | 9 | 9 | 5 | 4 | 4 | 4 |
| Aliivibrio | 3 | 9 | 4 | 3 | 1 | 1 | 1 | 1 | 1 | 1 | 1 | 1 | 1 | 1 |
| Chloroflexaceae | 3 | 23 | 14 | 10 | 6 | 4 | 2 | 2 | 1 | 1 | 1 | 1 | 1 | 1 |
| Micrococcaceae | 3 | 7 | 5 | 3 | 1 | 1 | 1 | 1 | 1 | 0 | 0 | 0 | 0 | 0 |
| Methylobacterium | 3 | 42 | 23 | 16 | 9 | 5 | 4 | 3 | 3 | 1 | 1 | 1 | 1 | 0 |
| Salmonella enterica subsp. enterica | 3 | 23 | 15 | 13 | 12 | 10 | 3 | 3 | 3 | 3 | 3 | 3 | 3 | 3 |
| Rhodospirillales | 3 | 10 | 3 | 1 | 1 | 0 | 0 | 0 | 0 | 0 | 0 | 0 | 0 | 0 |
| Nectriaceae | 3 | 9 | 8 | 7 | 5 | 5 | 5 | 4 | 3 | 3 | 0 | 0 | 0 | 0 |
| Campylobacter jejuni | 3 | 30 | 23 | 17 | 1 | 0 | 0 | 0 | 0 | 0 | 0 | 0 | 0 | 0 |
| Brassicales | 3 | 0 | 0 | 0 | 0 | 0 | 0 | 0 | 0 | 0 | 0 | 0 | 0 | 0 |
| Buchnera aphidicola | 3 | 2 | 1 | 1 | 0 | 0 | 0 | 0 | 0 | 0 | 0 | 0 | 0 | 0 |
| Thermotogaceae | 3 | 30 | 18 | 12 | 9 | 5 | 4 | 4 | 4 | 4 | 4 | 4 | 4 | 4 |
| Bradyrhizobiaceae | 3 | 42 | 29 | 26 | 9 | 7 | 7 | 7 | 7 | 2 | 1 | 1 | 1 | 0 |
| Thermoanaerobacteraceae | 3 | 12 | 5 | 1 | 1 | 1 | 1 | 1 | 0 | 0 | 0 | 0 | 0 | 0 |
| Hypocreaceae | 3 | 5 | 3 | 2 | 1 | 1 | 0 | 0 | 0 | 0 | 0 | 0 | 0 | 0 |
| Thermoproteaceae | 3 | 12 | 7 | 6 | 4 | 3 | 1 | 1 | 1 | 1 | 1 | 1 | 1 | 1 |
| Frankineae | 3 | 4 | 1 | 0 | 0 | 0 | 0 | 0 | 0 | 0 | 0 | 0 | 0 | 0 |
| Candida | 3 | 2 | 1 | 1 | 1 | 1 | 1 | 0 | 0 | 0 | 0 | 0 | 0 | 0 |
| Escherichia coli | 3 | 34 | 30 | 25 | 22 | 22 | 20 | 19 | 17 | 16 | 15 | 15 | 14 | 14 |
| Peptococcaceae | 3 | 25 | 12 | 6 | 0 | 0 | 0 | 0 | 0 | 0 | 0 | 0 | 0 | 0 |
| Helicobacter | 3 | 6 | 2 | 2 | 1 | 1 | 1 | 1 | 1 | 1 | 1 | 1 | 1 | 0 |
| pseudomallei group | 3 | 29 | 27 | 22 | 14 | 10 | 9 | 9 | 6 | 6 | 6 | 6 | 6 | 5 |
| Sordariales | 3 | 6 | 3 | 3 | 2 | 2 | 1 | 1 | 1 | 1 | 1 | 1 | 0 | 0 |
| Halobacteriaceae | 3 | 103 | 66 | 44 | 36 | 21 | 16 | 13 | 1 | 1 | 0 | 0 | 0 | 0 |
| Yersinia | 3 | 50 | 39 | 28 | 25 | 23 | 19 | 16 | 17 | 17 | 17 | 16 | 16 | 5 |
| Acinetobacter | 3 | 8 | 7 | 8 | 8 | 4 | 4 | 2 | 2 | 2 | 2 | 2 | 1 | 1 |
| Bacillus cereus group | 3 | 84 | 78 | 51 | 48 | 47 | 33 | 33 | 33 | 33 | 28 | 18 | 7 | 4 |
| Saccharomyces | 3 | 14 | 10 | 9 | 5 | 5 | 4 | 4 | 4 | 4 | 4 | 4 | 4 | 4 |
| Clostridium botulinum | 3 | 78 | 41 | 34 | 33 | 26 | 15 | 8 | 8 | 8 | 8 | 7 | 7 | 4 |
| Amoebozoa | 3 | 8 | 5 | 3 | 2 | 2 | 2 | 0 | 0 | 0 | 0 | 0 | 0 | 0 |
| Desulfuromonadales | 3 | 8 | 5 | 2 | 2 | 2 | 2 | 0 | 0 | 0 | 0 | 0 | 0 | 0 |
| Neisseria | 3 | 11 | 8 | 4 | 2 | 1 | 1 | 1 | 1 | 1 | 1 | 1 | 1 | 0 |
| Agaricomycetes | 3 | 22 | 14 | 6 | 2 | 2 | 1 | 0 | 0 | 0 | 0 | 0 | 0 | 0 |
| Haemophilus | 3 | 1 | 1 | 1 | 1 | 1 | 1 | 1 | 1 | 1 | 1 | 0 | 0 | 0 |
| Synechococcus | 3 | 8 | 14 | 8 | 7 | 6 | 1 | 0 | 0 | 0 | 0 | 0 | 0 | 0 |
| Chromatiales | 3 | 12 | 4 | 3 | 1 | 1 | 1 | 1 | 1 | 1 | 0 | 0 | 0 | 0 |
| Corynebacterium | 3 | 15 | 10 | 6 | 3 | 2 | 0 | 0 | 0 | 0 | 0 | 0 | 0 | 0 |
| Pucciniomycotina | 3 | 1 | 1 | 0 | 0 | 0 | 0 | 0 | 0 | 0 | 0 | 0 | 0 | 0 |
| Brucella | 3 | 10 | 7 | 7 | 7 | 3 | 3 | 2 | 2 | 2 | 0 | 0 | 0 | 0 |
| Lactococcus lactis | 3 | 4 | 3 | 2 | 2 | 1 | 1 | 1 | 1 | 1 | 1 | 1 | 1 | 0 |
| Actinobacillus | 3 | 2 | 0 | 0 | 0 | 0 | 0 | 0 | 0 | 0 | 0 | 0 | 0 | 0 |
| Streptococcus pyogenes | 3 | 30 | 17 | 13 | 11 | 9 | 6 | 6 | 6 | 4 | 3 | 3 | 2 | 2 |
| Shewanella | 3 | 64 | 34 | 27 | 15 | 17 | 15 | 11 | 8 | 8 | 7 | 7 | 9 | 9 |
| Nostocaceae | 3 | 54 | 19 | 15 | 10 | 6 | 4 | 4 | 3 | 3 | 2 | 2 | 2 | 2 |
| Methanococcus | 3 | 22 | 17 | 13 | 11 | 6 | 6 | 5 | 5 | 1 | 1 | 1 | 1 | 1 |
| Sulfolobaceae | 3 | 29 | 14 | 6 | 5 | 4 | 4 | 4 | 4 | 3 | 3 | 2 | 2 | 2 |
| Legionellales | 3 | 13 | 8 | 4 | 3 | 2 | 2 | 2 | 1 | 1 | 1 | 1 | 1 | 1 |
| Poaceae | 3 | 4 | 3 | 3 | 3 | 3 | 2 | 2 | 2 | 2 | 2 | 2 | 2 | 2 |
| Mamiellales | 3 | 13 | 10 | 8 | 5 | 2 | 2 | 2 | 2 | 1 | 1 | 1 | 0 | 0 |
| Burkholderia cepacia complex | 3 | 27 | 26 | 19 | 12 | 9 | 8 | 8 | 5 | 5 | 5 | 5 | 5 | 2 |
| Porphyromonadaceae | 3 | 3 | 1 | 1 | 1 | 1 | 1 | 1 | 0 | 0 | 0 | 0 | 0 | 0 |
| Myxococcaceae | 3 | 14 | 11 | 8 | 1 | 0 | 0 | 0 | 0 | 0 | 0 | 0 | 0 | 0 |
| Leptospira | 3 | 36 | 18 | 9 | 5 | 3 | 3 | 2 | 2 | 2 | 2 | 2 | 1 | 1 |
| Desulfurococcales | 3 | 9 | 4 | 2 | 2 | 0 | 0 | 0 | 0 | 0 | 0 | 0 | 0 | 0 |
| Shigella flexneri | 3 | 8 | 8 | 8 | 6 | 6 | 4 | 2 | 1 | 1 | 1 | 1 | 1 | 1 |
| Rhizobium | 3 | 17 | 10 | 7 | 6 | 6 | 1 | 1 | 1 | 1 | 1 | 1 | 1 | 1 |
| Plasmodium | 3 | 9 | 7 | 6 | 5 | 5 | 5 | 5 | 5 | 5 | 5 | 4 | 4 | 4 |
| Listeria | 3 | 22 | 14 | 12 | 10 | 9 | 4 | 3 | 2 | 2 | 1 | 1 | 1 | 1 |
| Nitrosomonadaceae | 3 | 13 | 8 | 5 | 5 | 3 | 1 | 1 | 1 | 1 | 1 | 1 | 0 | 0 |
| Francisella tularensis | 3 | 2 | 2 | 0 | 0 | 0 | 0 | 0 | 0 | 0 | 0 | 0 | 0 | 0 |
| Phyllobacteriaceae | 3 | 7 | 4 | 3 | 2 | 2 | 2 | 0 | 0 | 0 | 0 | 0 | 0 | 0 |
| Sordariomycetes incertae sedis | 3 | 0 | 0 | 0 | 0 | 0 | 0 | 0 | 0 | 0 | 0 | 0 | 0 | 0 |
| Thermococcaceae | 3 | 44 | 32 | 29 | 23 | 14 | 13 | 10 | 7 | 7 | 6 | 6 | 5 | 5 |
| Chlamydia | 3 | 2 | 1 | 1 | 1 | 1 | 1 | 1 | 1 | 1 | 1 | 0 | 0 | 0 |
| Aspergillus | 3 | 10 | 17 | 12 | 10 | 5 | 3 | 3 | 3 | 0 | 0 | 0 | 0 | 0 |
| Percomorpha | 3 | 5 | 2 | 1 | 1 | 1 | 0 | 0 | 0 | 0 | 0 | 0 | 0 | 0 |
| Candidatus Phytoplasma | 3 | 0 | 0 | 0 | 0 | 0 | 0 | 0 | 0 | 0 | 0 | 0 | 0 | 0 |
| melanogaster group | 3 | 3 | 3 | 3 | 3 | 3 | 1 | 1 | 1 | 1 | 1 | 1 | 1 | 1 |
| Chlorobium | 3 | 4 | 1 | 1 | 1 | 1 | 1 | 1 | 1 | 1 | 1 | 1 | 0 | 0 |
| Microbacteriaceae | 3 | 5 | 4 | 2 | 2 | 1 | 0 | 0 | 0 | 0 | 0 | 0 | 0 | 0 |
| Vibrio | 3 | 14 | 9 | 8 | 7 | 5 | 2 | 2 | 2 | 2 | 2 | 2 | 2 | 2 |
| Staphylococcus aureus | 3 | 15 | 13 | 12 | 9 | 6 | 6 | 6 | 6 | 5 | 5 | 5 | 5 | 4 |
| Hominidae | 3 | 5 | 4 | 3 | 2 | 1 | 1 | 1 | 1 | 1 | 1 | 0 | 0 | 0 |
| Bacteroides | 3 | 34 | 20 | 8 | 6 | 4 | 3 | 3 | 3 | 2 | 2 | 2 | 2 | 2 |
| Rhodobacteraceae | 3 | 25 | 26 | 15 | 11 | 9 | 6 | 2 | 2 | 1 | 0 | 0 | 0 | 0 |
| canis group | 3 | 2 | 0 | 0 | 0 | 0 | 0 | 0 | 0 | 0 | 0 | 0 | 0 | 0 |
| Comamonadaceae | 3 | 17 | 17 | 7 | 5 | 1 | 1 | 1 | 0 | 0 | 0 | 0 | 0 | 0 |
| unclassified Gammaproteobacteria | 3 | 1 | 1 | 1 | 0 | 0 | 0 | 0 | 0 | 0 | 0 | 0 | 0 | 0 |
| Deinococci | 3 | 8 | 5 | 4 | 3 | 3 | 3 | 2 | 2 | 2 | 2 | 2 | 2 | 2 |
| Pleosporales | 3 | 10 | 6 | 3 | 1 | 1 | 1 | 1 | 1 | 1 | 1 | 0 | 0 | 0 |
| Chromadorea | 3 | 11 | 5 | 5 | 5 | 4 | 3 | 3 | 3 | 3 | 3 | 3 | 3 | 3 |
| Desulfobacterales | 3 | 4 | 2 | 2 | 1 | 0 | 0 | 0 | 0 | 0 | 0 | 0 | 0 | 0 |
| Eimeriorina | 3 | 2 | 1 | 0 | 0 | 0 | 0 | 0 | 0 | 0 | 0 | 0 | 0 | 0 |
| Chlamydophila | 3 | 2 | 1 | 1 | 0 | 0 | 0 | 0 | 0 | 0 | 0 | 0 | 0 | 0 |
| Aquificales | 3 | 10 | 3 | 2 | 1 | 1 | 1 | 1 | 1 | 1 | 1 | 1 | 1 | 1 |
| Laurasiatheria | 3 | 2 | 2 | 3 | 4 | 3 | 3 | 2 | 2 | 1 | 1 | 1 | 0 | 0 |
| Pseudomonas aeruginosa group | 3 | 11 | 4 | 3 | 0 | 0 | 0 | 0 | 0 | 0 | 0 | 0 | 0 | 0 |
| spotted fever group | 3 | 5 | 8 | 1 | 1 | 1 | 0 | 0 | 0 | 0 | 0 | 0 | 0 | 0 |
| Bifidobacterium | 3 | 13 | 8 | 4 | 3 | 2 | 0 | 0 | 0 | 0 | 0 | 0 | 0 | 0 |
| Leuconostocaceae | 3 | 2 | 1 | 0 | 0 | 0 | 0 | 0 | 0 | 0 | 0 | 0 | 0 | 0 |
| Mycoplasma | 3 | 6 | 2 | 2 | 1 | 1 | 1 | 1 | 4 | 4 | 4 | 4 | 4 | 4 |
| Coccidioides | 3 | 6 | 3 | 1 | 1 | 1 | 1 | 1 | 0 | 0 | 0 | 0 | 0 | 0 |
| Methanosarcinaceae | 3 | 31 | 15 | 15 | 11 | 9 | 8 | 8 | 8 | 8 | 0 | 0 | 0 | 0 |
| stramenopiles | 3 | 48 | 18 | 8 | 4 | 2 | 1 | 0 | 0 | 0 | 0 | 0 | 0 | 0 |
| Xanthomonas | 3 | 41 | 35 | 25 | 17 | 15 | 13 | 11 | 10 | 8 | 8 | 8 | 2 | 2 |
| Flavobacteriaceae | 3 | 45 | 10 | 5 | 2 | 1 | 1 | 1 | 1 | 0 | 0 | 0 | 0 | 0 |
| Culicidae | 3 | 4 | 2 | 2 | 2 | 1 | 1 | 1 | 1 | 1 | 1 | 0 | 0 | 0 |
| Borrelia burgdorferi group | 3 | 15 | 7 | 3 | 3 | 2 | 2 | 2 | 2 | 2 | 2 | 2 | 2 | 2 |
| Sciurognathi | 3 | 0 | 0 | 0 | 0 | 0 | 0 | 0 | 0 | 0 | 0 | 0 | 0 | 0 |
| Thermoplasmatales | 3 | 39 | 23 | 16 | 14 | 7 | 7 | 7 | 4 | 4 | 4 | 3 | 3 | 3 |
| Sphingomonadales | 3 | 14 | 7 | 5 | 4 | 2 | 2 | 2 | 2 | 2 | 1 | 1 | 1 | 1 |
| Treponema | 3 | 2 | 2 | 1 | 1 | 0 | 0 | 0 | 0 | 0 | 0 | 0 | 0 | 0 |
